# Progression of the first stage of spontaneous labour: A prospective cohort study in two sub-Saharan African countries

**DOI:** 10.1371/journal.pmed.1002492

**Published:** 2018-01-16

**Authors:** Olufemi T. Oladapo, Joao Paulo Souza, Bukola Fawole, Kidza Mugerwa, Gleici Perdoná, Domingos Alves, Hayala Souza, Rodrigo Reis, Livia Oliveira-Ciabati, Alexandre Maiorano, Adesina Akintan, Francis E. Alu, Lawal Oyeneyin, Amos Adebayo, Josaphat Byamugisha, Miriam Nakalembe, Hadiza A. Idris, Ola Okike, Fernando Althabe, Vanora Hundley, France Donnay, Robert Pattinson, Harshadkumar C. Sanghvi, Jen E. Jardine, Özge Tunçalp, Joshua P. Vogel, Mary Ellen Stanton, Meghan Bohren, Jun Zhang, Tina Lavender, Jerker Liljestrand, Petra ten Hoope-Bender, Matthews Mathai, Rajiv Bahl, A. Metin Gülmezoglu

**Affiliations:** 1 UNDP/UNFPA/UNICEF/WHO/World Bank Special Programme of Research, Development and Research Training in Human Reproduction (HRP), Department of Reproductive Health and Research, World Health Organization, Geneva, Switzerland; 2 Department of Obstetrics and Gynaecology, College of Medicine, University of Ibadan, Ibadan, Nigeria; 3 Department of Obstetrics and Gynaecology, Makerere University, Kampala, Uganda; 4 Department of Social Medicine, Ribeirão Preto Medical School, University of São Paulo, Ribeirão Preto, São Paulo, Brazil; 5 Institute of Mathematical Science and Computing, University of São Paulo, São Carlos, São Paulo, Brazil; 6 Department of Obstetrics and Gynaecology, Mother and Child Hospital, Akure, Ondo State, Nigeria; 7 Department of Obstetrics and Gynaecology, Maitama District Hospital, Abuja, FCT, Nigeria; 8 Department of Obstetrics and Gynaecology, Mother and Child Hospital, Ondo, Ondo State, Nigeria; 9 Department of Obstetrics and Gynaecology, Asokoro District Hospital, Abuja, FCT, Nigeria; 10 Department of Obstetrics and Gynaecology, Nyanya General Hospital, Abuja, FCT, Nigeria; 11 Department of Obstetrics and Gynaecology, Karshi General Hospital, Abuja, FCT, Nigeria; 12 Department of Mother and Child Health Research, Institute for Clinical Effectiveness and Health Policy, Buenos Aires, Argentina; 13 Faculty of Health & Social Sciences, Bournemouth University, Bournemouth, United Kingdom; 14 Tulane University School of Public Health and Tropical Medicine, New Orleans, Louisiana, United States of America; 15 SAMRC/UP Maternal and Infant Health Care Strategies Unit, University of Pretoria, Pretoria, South Africa; 16 Jhpiego, an affiliate of Johns Hopkins University, Baltimore, Maryland, United States of America; 17 Women’s Health Research Unit, Queen Mary University of London, London, United Kingdom; 18 United States Agency for International Development, Bureau for Global Health, Washington D.C., United States of America; 19 Xinhua Hospital, Shanghai Jiao Tong University School of Medicine, Shanghai, China; 20 School of Nursing Midwifery & Social Work, University of Manchester, Manchester, United Kingdom; 21 Bill & Melinda Gates Foundation, Seattle, Washington, United States of America; 22 United Nations Population Fund, Geneva, Switzerland; 23 Department of Maternal, Newborn, Child and Adolescent Health, World Health Organization, Geneva, Switzerland; 24 Liverpool School of Tropical Medicine, Liverpool, United Kingdom; London School of Hygiene and Tropical Medicine, UNITED KINGDOM

## Abstract

**Background:**

Escalation in the global rates of labour interventions, particularly cesarean section and oxytocin augmentation, has renewed interest in a better understanding of natural labour progression. Methodological advancements in statistical and computational techniques addressing the limitations of pioneer studies have led to novel findings and triggered a re-evaluation of current labour practices. As part of the World Health Organization's Better Outcomes in Labour Difficulty (BOLD) project, which aimed to develop a new labour monitoring-to-action tool, we examined the patterns of labour progression as depicted by cervical dilatation over time in a cohort of women in Nigeria and Uganda who gave birth vaginally following a spontaneous labour onset.

**Methods and findings:**

This was a prospective, multicentre, cohort study of 5,606 women with singleton, vertex, term gestation who presented at ≤ 6 cm of cervical dilatation following a spontaneous labour onset that resulted in a vaginal birth with no adverse birth outcomes in 13 hospitals across Nigeria and Uganda. We independently applied survival analysis and multistate Markov models to estimate the duration of labour centimetre by centimetre until 10 cm and the cumulative duration of labour from the cervical dilatation at admission through 10 cm. Multistate Markov and nonlinear mixed models were separately used to construct average labour curves. All analyses were conducted according to three parity groups: parity = 0 (*n* = 2,166), parity = 1 (*n* = 1,488), and parity = 2+ (*n* = 1,952). We performed sensitivity analyses to assess the impact of oxytocin augmentation on labour progression by re-examining the progression patterns after excluding women with augmented labours. Labour was augmented with oxytocin in 40% of nulliparous and 28% of multiparous women. The median time to advance by 1 cm exceeded 1 hour until 5 cm was reached in both nulliparous and multiparous women. Based on a 95th percentile threshold, nulliparous women may take up to 7 hours to progress from 4 to 5 cm and over 3 hours to progress from 5 to 6 cm. Median cumulative duration of labour indicates that nulliparous women admitted at 4 cm, 5 cm, and 6 cm reached 10 cm within an expected time frame if the dilatation rate was ≥ 1 cm/hour, but their corresponding 95th percentiles show that labour could last up to 14, 11, and 9 hours, respectively. Substantial differences exist between actual plots of labour progression of individual women and the ‘average labour curves’ derived from study population-level data. Exclusion of women with augmented labours from the study population resulted in slightly faster labour progression patterns.

**Conclusions:**

Cervical dilatation during labour in the slowest-yet-normal women can progress more slowly than the widely accepted benchmark of 1 cm/hour, irrespective of parity. Interventions to expedite labour to conform to a cervical dilatation threshold of 1 cm/hour may be inappropriate, especially when applied before 5 cm in nulliparous and multiparous women. Averaged labour curves may not truly reflect the variability associated with labour progression, and their use for decision-making in labour management should be de-emphasized.

## Abstract 2

In a prospective cohort study, Olufemi Oladapo and colleagues investigated cervical dilation progression during the first stage of spontaneous labor in women from Nigeria and Uganda.

## Author summary

**Why was this study done?:**

- Dr Emmanuel Friedman’s studies on normal and abnormal labour progression have defined how labour should be managed since the mid-1950s until today. Although Friedman’s studies were conducted among pregnant women in the United States, the general belief that labour progression is the same in humans led to universal application of their findings, and the expectation that the cervix dilates by at least 1 cm/hour in all women during established labour.
- Since the early 2000s, however, researchers using new statistical methods to study labour found evidence to suggest that the patterns of labour progression as described by Friedman may not be accurate for the current generation of women giving birth. While these newer findings have informed changes in recommended labour practices in some settings, they have also generated a lot of controversy.
- As a result of persistent questions as to whether racial characteristics influence labour progression patterns, recent studies have been conducted among different populations, but not yet in any African population.

**What did the researchers do and find?:**

- We conducted an analysis of prospectively collected observational data of 5,606 women who presented in early labour (at or before 6 cm of cervical dilatation) following spontaneous labour onset and gave birth vaginally in 13 maternity hospitals in Nigeria and Uganda. None of these women experienced serious adverse outcomes for themselves or their babies.
- We applied advanced statistical and computational methods (survival analysis and Markov techniques) to determine how long it took the cervix to dilate by 1 cm from one level of dilatation to the next until full dilatation (10 cm) and how long it took the cervix to reach full dilatation based on the dilatation at the time of labour admission. We also used two separate methods to plot population average cervical dilatation time curves (labour curves) for the women in our sample.
- Contrary to the generally held view, we found that labour progressed more slowly in our study population than previously reported. On average, the rate of cervical dilatation was less than 1 cm/hour for some women until 5 cm of cervical dilatation was reached among those undergoing their first, second, or subsequent labours.
- Labour was very slow in some women throughout the first stage, including the early part of the period that is traditionally known as the ‘active phase’, when the ‘normal’ cervical dilatation rate is expected to be at least 1 cm/hour or faster. While on average the labour progression in first-time mothers was generally similar to their counterparts in the US, China, and Japan, there are also important differences in the slowest-yet-normal (95th percentile) group of women in our study population.

**What do these findings mean?:**

- The average labour curves derived from our study population are substantially different from those published from the pioneer work of Friedman. They also do not truly reflect the variations shown in the labour progression of individual women in our study.
- The application of population average labour curves could potentially misclassify women who are slowly but normally progressing as abnormal and therefore increase their chances of being subjected to unnecessary labour interventions. We propose that averaged labour progression lines or curves are not used for decision-making in the management of labour for individual women.
- As labour may not naturally accelerate in some women until a cervical dilatation of 5 cm is reached, labour practices to address perceived slow labour progression should not be routinely applied by clinicians until this threshold is achieved, provided the vital signs and other observations of the mother and baby are normal.
- In the absence of any problems other than a slower than expected cervical dilatation rate (i.e., 1 cm/hour) during labour, it is in the interest of the woman that expectant, supportive, and woman-centred labour care is continued.

## Introduction

From the mid-1950s until the 1980s, Dr Emmanuel Friedman published a series of landmark studies describing the patterns of labour progression in nulliparous and multiparous women [1–9]. The classic sigmoidal labour curve derived from his work has defined the fundamental basis of labour management for more than six decades. Although Friedman’s studies were limited to obstetric populations in the US, the general notion that the labour progression pattern is largely consistent in humans has led to universal application of their findings and the expectation that the cervix dilates by at least 1 cm/hour in all women. This long-held assumption was the basis for the introduction of ‘Active Management of Labour’ protocols by O’Driscoll and colleagues in the 1970s [10], to ‘normalize’ women’s labour patterns in accordance with the ‘1 cm/hour rule’. However, the escalating rates of unnecessary labour interventions over the last two decades, particularly oxytocin augmentation and cesarean section [11], have renewed interest in what constitutes normal labour progression.

Since the late 1990s and early 2000s, there is increasing evidence to suggest that the descriptions of the relationship between the duration of first stage of labour and cervical dilatation patterns and the definitions of labour dystocia as earlier described may not be appropriate [12–16]. Labour interventions such as induction, oxytocin augmentation, and epidural anaesthesia are now more common, while instrumental and breech vaginal births are becoming rare. The generation of women giving birth in contemporary practice is older, and with increasing body mass index and fetal weight.

In addition, newer research has taken advantage of methodological advancements in computational techniques to address the limitations of studying labour progression and constructing labour curves in the 1950s and 1960s [17]. While these advancements have led to novel findings and new guidance on labour care [18], they are also a subject of intense debate [19–21]. Suggestions that there may be racial and ethnic differences in labour progression patterns as a result of differences in pelvic configurations and sociocultural aspects have promoted research in different obstetric populations [22]. While contemporary labour curves have been published for white, Hispanic, and Asian obstetric populations [14–16], no modern labour curves exist for sub-Saharan African women.

As part of the WHO’s Better Outcomes in Labour Difficulty (BOLD) project, which aimed to develop an innovative and effective labour monitoring-to-action tool [23], we examined the patterns of labour progression in a prospective cohort of women in Nigeria and Uganda who gave birth vaginally without adverse birth outcomes following a spontaneous labour onset.

## Methods

### Ethics statement

Scientific and technical approval for this study was obtained from the Review Panel on Research Projects (RP2) of the UNDP/UNFPA/UNICEF/WHO/World Bank Special Program of Research, Development and Research Training in Human Reproduction (HRP), Department of Reproductive Health and Research, WHO. Ethical approval was obtained from the WHO Ethical Review Committee (protocol A65879), the Makerere University School of Health Sciences Research and Ethics Committee, Uganda (protocol #SHSREC REF 2014–058), University of Ibadan/University College Hospital Ethics Committee (UI/EC/14/0223), Federal Capital Territory Health Research Ethics Committee, Nigeria (protocol FHREC/2014/01/42/27-08-14), and Ondo State Government Ministry of Health Research Ethics Review Committee, Nigeria (AD 4693/160). The study was conducted according to the Declaration of Helsinki of the World Medical Association.

### Design, setting, and population

The WHO BOLD research project was primarily designed to identify the essential elements of labour monitoring that trigger the decision to use interventions aimed at preventing poor labour outcomes, with the aim of developing a new labour monitoring-to-action tool. The study protocol and detailed methodological considerations have been published elsewhere [23]. In brief, this was a prospective, multicentre, cohort study of women admitted for vaginal birth with single live fetuses during early first stage of labour across 13 hospitals in Nigeria and Uganda. This included women undergoing induction of labour and those with spontaneous labour onset who presented at cervical dilatation of ≤ 6 cm. Women with multiple pregnancies, gestational age less than 34 weeks, elective cesarean section, and those who were unwilling to participate or incapable of giving consent due to obstetric emergencies were excluded. 9,995 women (56.1%) out of 17,810 women who were screened in all hospitals during the study period met these inclusion criteria and participated in the study.

Participating hospitals had a minimum of 1,000 deliveries per year with stable access to cesarean section, augmentation of labour, and instrumental vaginal birth. Estimation of gestational age at birth was in accordance with individual institutional practices, which relied upon the woman’s first date of the last menstrual period in the majority of cases. Labour was managed by midwives or obstetric residents and/or obstetricians. Doppler fetal monitor was used to assess fetal vital status at hospital admission and for intermittent monitoring throughout labour. Labour management protocol, as well as the number and timing of pelvic examinations, were not standardized across participating institutions. None of the institutions subscribed to the ‘Active Management of Labour’ protocol during the study period. Although the partograph was a standard element in all labour protocols, adherence to its application for labour management during the study period varied widely across hospitals.

### Study procedures

Eligible women were recruited into the study between December 2014 and November 2015. From the medical record, trained research nurses prospectively extracted detailed information on sociodemographic, anthropometric, obstetric, and medical characteristics of study participants at hospital admission, multiple assessments for labour monitoring and interventions performed throughout the first and second stages of labour, and maternal and neonatal outcomes following labour. Attending staff were approached to complement medical records data when needed. Data collection was limited to the hospital stay of the mother and baby, and there was no follow-up after hospital discharge.

**Fig 1:**
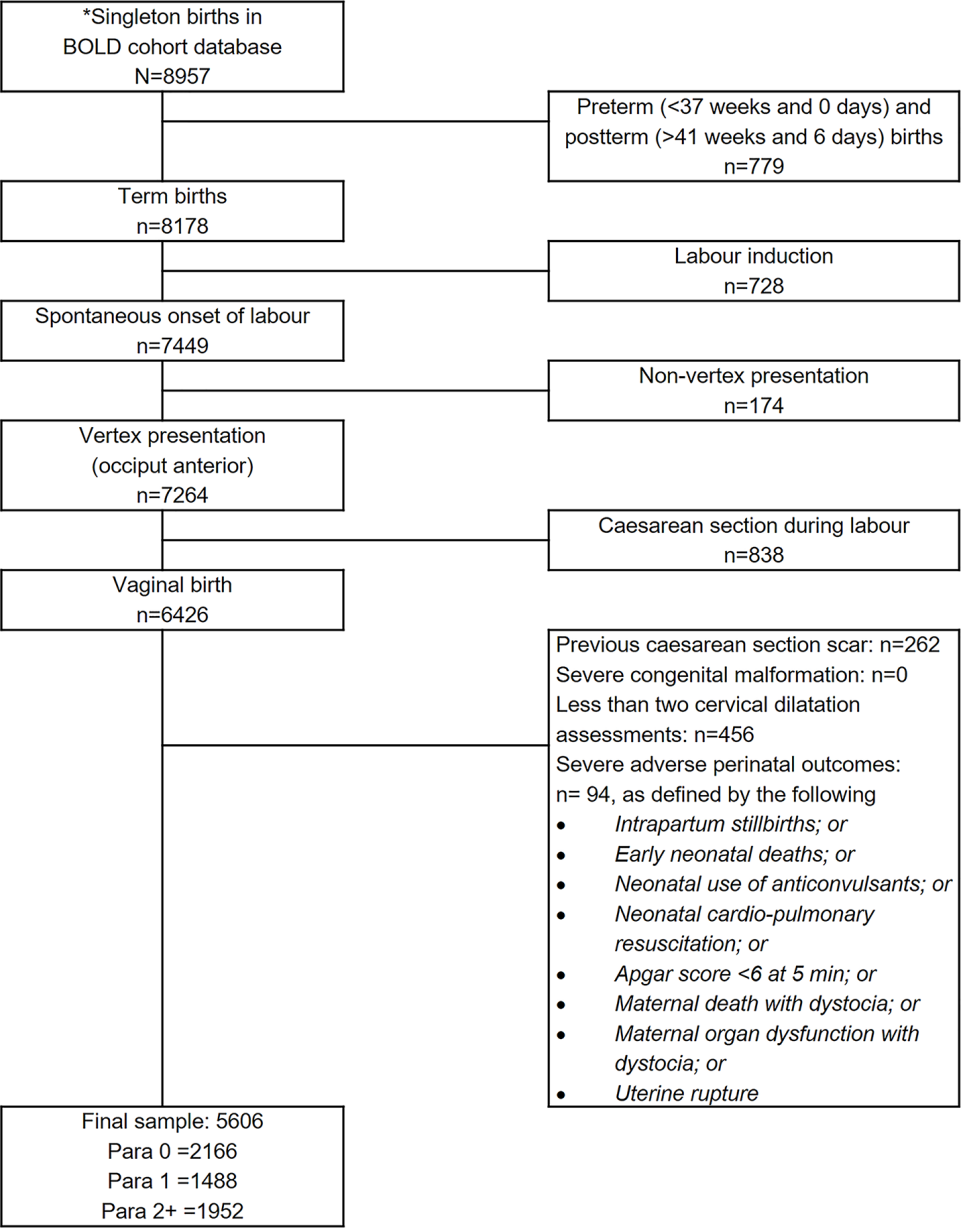
Sample selection flow chart. * Excluding significant outliers due to unusual rapidity, regression, or inconsistencies with time. BOLD, Better Outcomes in Labour Difficulty.

The current study used information on maternal baseline and admission characteristics, repeated assessments of cervical dilatation over time, maternal and neonatal characteristics throughout labour, and perinatal outcome data. This analysis was focused on describing the labour patterns of women without adverse birth outcomes and not on determining correlation to clinical outcomes (See S1 STROBE Checklist). From a total of 8,957 singleton births with consistent time records in the database, we restricted our analysis to examine labour progression to 5,606 women on the basis of the following inclusion criteria (Fig 1): term births (between 37 weeks and 0 days and 41 weeks and 6 days) with vertex presentation and spontaneous labour onset. We excluded women who had labour induction, previous uterine scar, or intrapartum cesarean section. To examine the labour patterns in women with normal perinatal outcomes, we excluded women whose labour resulted in severe adverse outcomes, which was defined as occurrence of any of the following: stillbirth, early neonatal death, neonatal use of anticonvulsant, neonatal cardiopulmonary resuscitation, 5-minute Apgar score < 6, maternal death or organ dysfunction associated with labour dystocia, or uterine rupture. Furthermore, we excluded women who gave birth to neonates with severe congenital malformation and those with fewer than two cervical dilatation assessments during the first stage of labour (since a single data point cannot be used to generate a labour pattern for the individual woman).

### Data analysis

We grouped women in the selected sample into three parity groups (0, 1, and 2+) to explore any differences in labour patterns according to parity. We used two independent approaches to analyse labour progression patterns and construct average labour curves for the selected sample. In the first approach, we performed survival analyses to estimate the time it took to progress from one level of cervical dilatation to the next (called ‘sojourn time’) (i.e., from 3 to 4 cm, 4 to 5 cm, 5 to 6 cm, until full dilatation [10 cm]). We used both complete (where available) and interval-censored times to estimate the distribution of times for progression from one integer centimetre of dilatation to the next, with an assumption that the labour data are log-normally distributed. Based on this model, the median, 5th, and 95th percentiles were calculated. We used the same approach to derive the cumulative duration of labour for women presenting at different cervical dilatations (3 cm, 4 cm, 5 cm, and 6 cm) to evaluate any potential differences in the patterns of labour progression. To illustrate the ‘slowest-yet-normal’ labour patterns, we plotted the 95th percentiles for the cumulative duration of labour based on the cervical dilatation at admission. To construct average labour curves, we applied a nonlinear mixed model that best fit our data instead of polynomial models used by previous authors [12–16, 24]. We expressed cervical dilatation for subject *i* in time *j* (y_*ij*_) as a function of time (t_ij_) according to the following three-parameter logistic growth model:

$$y_{ij}=\beta_{0}+\frac{\beta_{1}}{1+\exp\left(-(t_{ij}-(\beta_{2}+b_{i}))\right)}$$

in which β_0_ is dilatation value when t_ij_ → −∞, β_1_ is the asymptotic curve height, and β_2_ is the inflection point and at this time value when the dilatation reaches half of its height. For simplicity, we estimated β_0_, β_1_ as fixed effects and included the random term b_i_ in the inflection point and assumed that this term follows a normal distribution, i.e., $b_{i}∼N(0,\sigma_{b}^{2})$. Given that women in this analysis entered the cervical dilatation time curve at different dilatations but all ended at full dilatation (10 cm), the starting point (time = 0) on the x-axis was set at full dilatation (10 cm), which was reached by all women in the sample and then calculated backwards (e.g., 1 hour before 10 cm becomes −1 hour and so on). This x-axis (time) was then reverted to a positive value. For example, instead of −12 → 0 hours, it became 0 → 12 hours. We used R-Cran version 3.2 for these statistical analyses [25].

In the second approach, we applied a multistate Markov modelling technique to examine the labour progression patterns in the same sample. This mathematical modelling technique from matrix algebra describes the transitions that a cohort of individuals make among a number of mutually exclusive and exhaustive health states during a series of short time intervals [26]. As cervical dilatation progression is a state- and time-related phenomenon during a period ranging from labour onset through to full cervical dilatation and birth of the baby (i.e., there is a finite set of states), the labour process can be considered a mathematical model that is suitable for the application of multistate Markov modelling. We therefore represented the sequence of labour progress as states based on every observed centimetre from 2 to 10 cm until birth of the baby—the ‘absorbing state’, as illustrated in S1 Fig. At a time *t*, the woman is in state *S*(*t*). The model was designed as a progressive unidirectional model, which only allows a choice of a way out of a particular state, but once a woman has left a state she cannot return. The next state to which a woman moves and the time of the change are governed by a set of transition intensities for each pair of states *r* and *s*. The transition intensity represents the instantaneous likelihood of moving from state *r* to state *s*. The full set of intensities for the system form the matrix *Q*.

A Markov process is based on the transition matrix with a probability structure *P*(*u*, *t* + *u*). The (*r*, *s*) entry (the elements of entire matrix) of *P*(*u*, *t* + *u*), is the probability of being in state *s* at a time *t* + *u*, given the state at time *u* is *r*. *P*(*u*, *t* + *u*) is calculated in terms of *Q*. Assuming that the transition intensity matrix *Q* is constant over the interval (*u*, *t* + *u*), as in a time-homogeneous process, *P*(*u*, *t* + *u*) = *P*(*t*) and the equations are solved by the matrix exponential of *Q* scaled by the time interval, *P*(*t*) = *Exp*(*tQ*) (S1 Fig). We used msm package for R Project programming environment to fit the multistate Markov model [26]. We generated random observations of cervical dilatation based on the transition matrix *P*(*t*) for the entire duration of labour (S2 Fig) to derive average labour curves according to parity and calculated the median, 5th, and 95th percentiles of sojourn times and cumulative duration of labour according to cervical dilatation at admission.

In order to assess the influence of oxytocin augmentation on the described labour patterns, we applied the survival analyses and multistate Markov models to perform sensitivity analyses comparing labour progression patterns of all women with that of a population excluding women with oxytocin augmentation (i.e., our entire study population versus study population excluding women with augmented labours).

The plan for the above survival analyses was first presented at an expert meeting convened by the WHO in November 2016, following which the analyses were started. In February 2017, after a review of the preliminary results of these analyses, the WHO study-coordinating unit requested an independent application of multistate Markov models to the same sample of women in order to determine whether the findings are consistent between the two analytical approaches. From June to July 2017, sensitivity analyses were conducted using the two analytical approaches to assess the influence of oxytocin augmentation on the described labour patterns for the study population, following the suggestions of the BOLD project technical advisory group and study co-authors.

## Results

### Baseline characteristics, labour observations, and interventions

A total of 5,606 women were included in these analyses. Table 1 presents the characteristics of these women by parity. In the selected sample, 54.7% of the women were from Uganda and 45.3% were from Nigeria. Nulliparous women were younger than the multiparous women, constituted over a third of the study sample, and were evenly balanced between the two countries. There was a slight increase in maternal body mass index at birth as parity increased. At labour admission, spontaneous rupture of the membranes had occurred in a quarter of nulliparous women and in about one-fifth of multiparous women. The cervix was well effaced (thin or very thin) in half of the nulliparous and in slightly higher proportions in the multiparous groups. Median cervical dilatation was 4 cm, and the fetal head was not engaged in over 90% of women in all parity groups. There was no caput succedaneum or moulding in over 99% of the women at the time of admission.

**Table 1:** Labour characteristics and interventions by parity.

Demographic characteristics	Parity = 0	Parity = 1	Parity = 2+
Study population: *N* = 5,606	2,166	1,488	1,952
Country			
*Nigeria*	1,102 (50.88)	645 (43.35)	793 (40.62)
*Uganda*	1,064 (49.12)	843 (56.65)	1,159 (59.38)
Age: years, mean (SD)	25.12 (4.17)	27.14 (4.05)	30.98 (4.64)
Maternal height: cm, mean (SD)	159.88 (6.76)	159.96 (6.57)	160.43 (6.67)
Maternal weight at delivery: kg, mean (SD)	71.84 (11.59)	73.82 (12.35)	76.37 (12.55)
Maternal BMI at delivery: mean (SD)	28.09 (4.12)	28.86 (4.51)	29.66 (4.53)
**Labour admission observations**			
Amniotic membranes status: *N* (%)			
*Intact*	1,630 (75.25)	1,189 (79.91)	1,530 (78.38)
*Ruptured*	534 (24.65)	295 (19.83)	420 (21.52)
*Unknown*	2 (0.09)	4 (0.27)	2 (0.10)
Cervix effacement: *N* (%)			
*Thick (<30%)*	338 (15.60)	213 (14.31)	299 (15.32)
*Medium (up to 50%)*	745 (34.40)	422 (28.36)	585 (29.97)
*Thin (up to 80%)*	918 (42.38)	737 (49.53)	921 (47.18)
*Very thin (>80%)*	160 (7.39)	110 (7.39)	142 (7.27)
*Unknown*	5 (0.23)	6 (0.40)	5 (0.26)
Cervical dilatation: cm, median (10th, 90th percentiles)	4 (2, 6)	4 (2, 6)	4 (2, 6)
Fetal station: *N* (%)			
*Above ischial spine*	1,591 (73.45)	1,056 (70.97)	1,382 (70.80)
*At ischial spine*	438 (20.22)	316 (21.24)	389 (19.93)
*Below ischial spine*	136 (6.28)	112 (7.53)	173 (8.86)
*Unknown*	1 (0.05)	4 (0.27)	8 (0.41)
Caput succedaneum: *N* (%)			
*None*	2,158 (99.60)	1,486 (99.90)	1,949 (99.80)
*Mild*	7 (0.30)	1 (0.10)	3 (0.20)
*Moderate*	1 (0.00)	0 (0.00)	0 (0.00)
*Severe*	0 (0.00)	0 (0.00)	0 (0.00)
*Unknown*	0 (0.00)	1 (0.07)	0 (0.00)
Moulding: *N* (%)			
*0 (none)*	2,151 (99.30)	1,479 (99.50)	1,942 (99.50)
*1+ (first degree)*	13 (0.60)	8 (0.50)	10 (0.50)
*2+ (second degree)*	2 (0.10)	0 (0.00)	0 (0.00)
*3+ (third degree)*	0 (0.00)	0 (0.00)	0 (0.00)
*Unknown*	0 (0.00)	1 (0.07)	0 (0.00)
**Intrapartum interventions and observations**			
Total number of vaginal examinations in first stage: median (10th, 90th percentiles)	3 (2, 5)	3 (2, 4)	3 (2, 4)
Augmentation with oxytocin infusion: *N* (%)	866 (40.00)	444 (29.80)	522 (26.70)
Labour analgesia: *N* (%)			
*IV/IM Opioid*	69 (3.20)	22 (1.50)	17 (0.90)
*Epidural*	0 (0.00)	1 (0.10)	0 (0.00)
*Spinal*	1 (0.00)	0 (0.00)	0 (0.00)
*Other*	31 (1.40)	17 (1.10)	21 (1.10)
*Combined*	0 (0.00)	1 (0.10)	0 (0.00)
Presence of a labour companion*: *N* (%)			
*0*	1,053 (48.61)	661 (44.42)	808 (41.39)
*1*	304 (14.04)	300 (20.16)	414 (21.21)
*2*	371 (17.13)	276 (18.55)	400 (20.49)
*≥3*	429 (19.81)	240 (16.13)	316 (16.19)
*Unknown*	9 (0.42)	11 (0.74)	14 (0.72)
Oral fluid intake*: *N* (%)			
*0*	674 (31.12)	453 (30.44)	556 (28.48)
*1*	489 (22.58)	451 (30.31)	587 (30.07)
*2*	531 (24.52)	343 (23.05)	464 (23.77)
*≥3*	463 (21.38)	230 (15.46)	328 (16.80)
*Unknown*	9 (0.42)	11 (0.74)	17 (0.87)
Oral food intake*: *N* (%)			
*0*	1,698 (78.39)	1,187 (79.77)	1,561 (79.97)
*1*	273 (12.60)	207 (13.91)	255 (13.06)
*2*	118 (5.45)	52 (3.49)	92 (4.71)
*≥3*	67 (3.09)	29 (1.95)	27 (1.38)
*Unknown*	10 (0.46)	13 (0.87)	17 (0.87)
Caput succedaneum**: *N* (%)			
*None*	1,911 (88.23)	1,368 (91.94)	1,814 (92.93)
*Mild*	202 (9.33)	102 (6.85)	113 (5.79)
*Moderate*	44 (2.03)	10 (0.67)	16 (0.82)
*Severe*	0 (0.00)	0 (0.00)	0 (0.00)
*Unknown*	9 (0.42)	8 (0.54)	9 (0.46)
Moulding**: *N* (%)			
*0 (none)*	1,758 (81.16)	1,219 (81.92)	1,598 (81.86)
*1+ (first degree)*	332 (15.33)	225 (15.12)	312 (15.98)
*2+ (second degree)*	64 (2.95)	35 (2.35)	32 (1.64)
*3+ (third degree)*	2 (0.10)	1 (0.10)	1 (0.10)
*Unknown*	10 (0.46)	8 (0.54)	9 (0.46)
**Birth outcomes**			
Mode of birth: *N* (%)			
*Spontaneous vaginal birth (without episiotomy)*	849 (39.20)	1,218 (81.90)	1,798 (92.10)
*Spontaneous vaginal birth (with episiotomy)*	1,255 (57.90)	250 (16.80)	145 (7.40)
*Operative vaginal birth (forceps or vacuum)*	62 (2.90)	20 (1.30)	9 (0.50)
Gestational age at birth: weeks, mean (SD)	38.74 (1.11)	38.74 (1.11)	38.77 (1.10)
Birth weight: g, mean (SD)	3,139.72 (404.22)	3,277.48 (409.12)	3,348.28 (438.91)

*Frequency of observations during intrapartum assessments

**Most ‘severe’ observation during intrapartum assessments

In terms of labour interventions, 40% of nulliparous women received oxytocin infusion for labour augmentation, compared with 28% of multiparous women. The median number of vaginal examinations per woman throughout first stage was 3. Presence of a labour companion was observed at least on one occasion in more than half of the women and on two or more occasions in at least a third. While over two-thirds of the women were observed to have taken oral fluids at least once during labour, less than half of them were observed to have done so two or more times. In comparison, oral feedings were observed less frequently, although the observed pattern was similar across parity groups. Severe caput succedaneum and third-degree moulding of the fetal head were rarely seen in any of the parity groups. Labour analgesia and operative vaginal birth were used in less than 2% in the study population; a reflection of the current clinical practices in the study hospitals. While the gestational age at birth was similar across the parity groups, there was an average of a 100-g increase in birth weight with increasing parity.

### Labour progression patterns (all women)

Table 2 presents the detailed analyses of labour progression based on the two analytical approaches and compares these with the findings of Zhang et al. [14]. The table shows that, based on survival analyses, the median time for the cervix to dilate by 1 cm was longer than the generally accepted limit of 1 hour until a cervical dilatation of 5 cm was achieved in nulliparous women and until 5 cm was achieved in multiparous women. In all parity groups, the median rate of progression doubles as the cervix reaches 6 cm with a median time shorter than 1 hour. Labour progression afterwards escalated more rapidly as it advanced towards 10 cm in all parity groups. Likewise, multistate Markov modelling shows that the median time needed to advance by 1 cm was more than 1 hour until 5 cm was achieved in both nulliparous and multiparous women, and labour progression became more rapid from 7 cm. The distribution of data from both analysis methods show a wide variability around the median for each level of advancement, though this was more pronounced in the survival analyses data. The 95th percentiles of the distribution of sojourn times indicate that labour could progress much more slowly for some women and still result in vaginal birth without adverse birth outcomes. The data show that it was not unusual for nulliparous women to spend more than 7 hours to advance from 4 to 5 cm and over 3 hours to advance from 5 to 6 cm. For some women, the 95th percentile data suggest that throughout the first stage of labour, it took more than 1 hour for cervical dilatation to advance by 1 cm irrespective of the parity groups. The table also shows that the pattern of median times to advance from early to advanced first stage of labour is largely consistent with the findings of Zhang et al. [14], although our 95th percentiles show even wider variability.

**Table 2:** Duration of labour from one level of cervical dilatation to the next by parity and analysis method.

**Parity**	**Parity = 0**			**Parity = 1**			**Parity = 2+**		
Study	Current study	Current study	Zhang et al. [14]	Current study	Current study	Zhang et al. [14]	Current study	Current study	†Zhang et al. [14]
*N*	2,166	2,166	25,624	1,488	1,488	16,755	1,952	1,952	16,219
Cervical dilatation	Survival analysis†	Markov model	Survival analysis‡	Survival analysis†	Markov model	Survival analysis‡	Survival analysis†	Markov model	Survival analysis‡
3–4 cm	2.82 (0.60, 13.33)	1.83 (0.08, 8.17)	1.8 (8.1)	2.42 (0.41; 14.18)	1.92 (0.08, 8.33)	NA	2.35 (0.31; 17.85)	2.17 (0.08, 9.75)	NA
4–5 cm	1.72 (0.38, 7.83)	1.58 (0.08, 7.08)	1.3 (6.4)	1.37 (0.25; 7.65)	1.42 (0.08, 6.42)	1.4 (7.3)	1.18 (0.17; 8.05)	1.50 (0.08, 6.5)	1.4 (7.0)
5–6 cm	1.19 (0.23, 6.17)	0.83 (0.00, 3.83)	0.8 (3.2)	0.79 (0.13; 4.95)	0.83 (0.00, 3.58)	0.8 (3.4)	0.79 (0.10; 6.24)	0.75 (0.00, 3.33)	0.8 (3.4)
6–7 cm	0.66 (0.09, 4.92)	0.92 (0.00, 4.25)	0.6 (2.2)	0.33 (0.03; 3.67)	0.75 (0.00, 3.50)	0.5 (1.9)	0.31 (0.03; 3.29)	0.83 (0.00, 3.58)	0.4 (1.2)
7–8 cm	0.25 (0.02, 3.10)	0.58 (0.00, 2.50)	0.5 (1.6)	0.09 (0.00; 2.69)	0.42 (0.00, 1.83)	0.4 (1.3)	0.17 (0.01; 2.44)	0.33 (0.00, 1.50)	0.3 (0.9)
8–10 cm	0.87 (0.18, 4.19)	0.75 (0.00, 3.33)	0.5 (1.4)*; 0.5 (1.8)§	0.64 (0.11; 3.56)	0.67 (0.00, 2.92)	0.3 (1.0)*; 0.3 (0.9)§	0.68 (0.12; 3.77)	0.50 (0.00, 2.50)	0.3 (0.8)*; 0.3 (1.6)§

Current study data reported as median hours (5th, 95th percentiles).

Zhang et al. data reported as median hours (95th percentile).

† Survival analysis with complete and interval-censored values

‡ Survival analysis with interval-censored regression

* 8–9 cm.

§ 9–10 cm.

**Fig 2:**
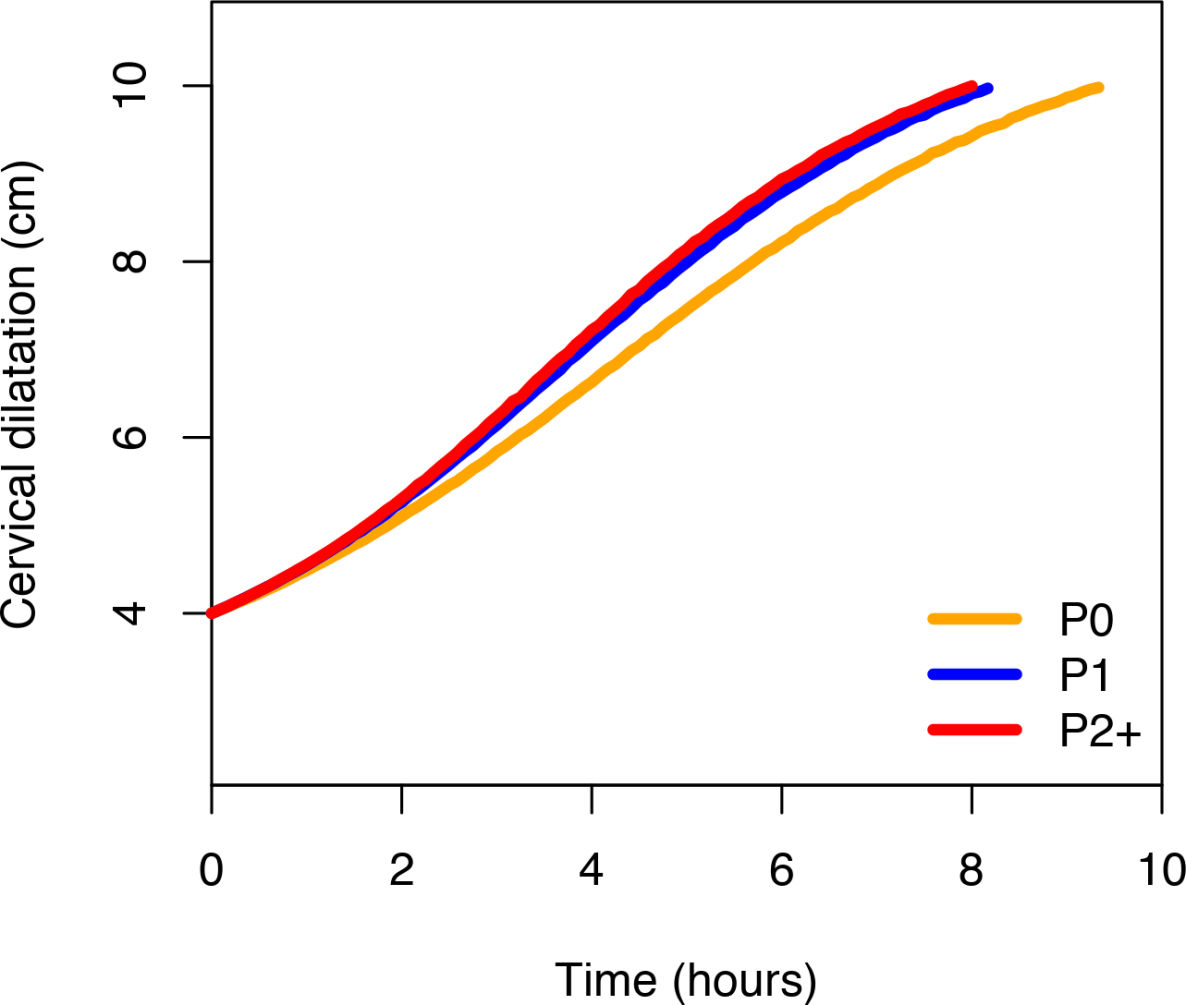
Average labour curves by parity based on multistate Markov models. P0, nulliparous women; P1, parity = 1 women; P2+, parity = 2+ women.

Fig 2 shows that the ‘average labour curves’ derived from multistate Markov models for both nulliparous and multiparous women progressed gradually from 4 cm with fairly linear trajectories as they advanced towards 10 cm. The slopes of the curves for multiparous women were steeper than that of the nulliparous women.

The nonlinear mixed models, however, produced smooth labour curves for both nulliparous and multiparous women, which proceeded gradually with a slight upward inclination from around 5 cm and no clear inflection points through 10 cm (S3 Fig). Inflection points appear outside the normal range of observations. Within the range of observed data for cervical dilatation, the curves appear to accelerate from 5 cm, with steeper slopes as they advanced towards 10 cm in multiparous compared to nulliparous women.

S1 Video, S2 Video, S3 Video, and S4 Video are video displays comparing actual plots of cervical dilatation pattern of individual women (starting from 4 cm) with (1) the average labour curves constructed from our study population and (2) the 1 cm/hour alert line of the partograph. The videos show that a substantial proportion of nulliparous and multiparous women crossed the 1 cm/hour alert line as they progressed during labour. The videos also show that substantial differences exist between actual plots of labour progression for individual women and the population average curves.

Table 3 shows the cumulative duration of labour from the cervical dilatation observed at admission (e.g., at 3 cm, 4 cm, 5 cm, or 6 cm) to the next centimetre until 10 cm. The table shows that the median times estimated by the two analysis methods are mostly consistent but also have wide variability in data distribution expressed by their corresponding 5th and 95th percentiles. The rapid progression of cervical dilatation in advanced labour as shown by the sojourn times (in Table 2) is also expressed by the progressively shorter cumulative duration of labour as cervical dilatation on admission increased from 4 to 6 cm. The median rates of ‘linear dilatation’ increased from 1 cm/hour for nulliparous women admitted at 4 cm to 1.3 cm/hour for those admitted at 6 cm. While the median times for nulliparous women admitted at 4, 5, and 6 cm to achieve full dilatation were within the same time frame for dilatation progressing at ≥1 cm/hour, their 95th percentiles show that it was not uncommon to have labours lasting up to 14, 11, and 9 hours in the same categories of women, respectively. The observed cumulative duration of labour in women arriving in labour before 4 cm shows that some of these women did not deliver vaginally until almost 24 hours after admission. The overall patterns are similar for multiparous women, although the medians and their corresponding 95th percentiles were generally shorter than for nulliparous women.

**Table 3:** Cumulative duration of labour in Para 0, 1, and 2+ based on the cervical dilatation at admission.

**Parity = 0**								
	Survival analysis	Markov model	Survival analysis	Markov model	Survival analysis	Markov model	Survival analysis	Markov model
Cervical dilatation at:	Adm. at 3 cm (*N* = 249)	Adm. at 3 cm (*N* = 249)	Adm. at 4 cm (*N* = 715)	Adm. at 4 cm (*N* = 715)	Adm. at 5 cm (*N* = 316)	Adm. at 5 cm (*N* = 316)	Adm. at 6 cm (*N* = 322)	Adm. at 6 cm (*N* = 322)
Adm. to 3 cm								
Adm. to 4 cm	2.76 (0.58, 13.10)	1.83 (0.08, 8.17)						
Adm. to 5 cm	4.49 (1.17, 17.17)	4.25 (0.83, 12.08)	1.71 (0.37, 7.96)	1.58 (0.08, 7.08)				
Adm. to 6 cm	5.65 (1.65, 19.40)	5.58 (1.67, 13.67)	3.02 (0.86, 10.60)	2.92 (0.58, 8.92)	1.29 (0.28, 6.05)	0.83 (0.00, 3.83)		
Adm. to 7 cm	6.50 (1.99, 21.26)	7.08 (2.50, 15.50)	4.15 (1.41, 12.19)	4.42 (1.33, 10.83)	2.10 (0.51, 8.66)	2.25(0.42, 6.42)	0.78 (0.11, 5.46)	0.92 (0.00, 4.25)
Adm. to 8 cm	7.19 (2.34, 22.14)	7.92 (3.17, 16.58)	4.97 (1.87, 13.24)	5.33 (1.92, 12.00)	3.06 (0.93, 10.14)	3.08 (0.92, 7.50)	1.76 (0.42, 7.44)	1.83 (0.33, 5.50)
Adm. to 10 cm	8.37 (2.98, 23.51)	9.08 (4.00, 17.83)	5.92 (2.42, 14.48)	6.50 (2.67, 13.25)	4.30 (1.64, 11.30)	4.25 (1.58, 9.17)	2.86 (0.88, 9.30)	3.00 (0.92, 7.25)
**Parity = 1**								
Cervical dilatation at:	Adm. at 3 cm (*N* = 164)	Adm. at 3 cm (*N* = 164)	Adm. at 4 cm (*N* = 491)	Adm. at 4 cm (*N* = 491)	Adm. at 5 cm (*N* = 292)	Adm. at 5 cm (*N* = 292)	Adm. at 6 cm (*N* = 320)	Adm. at 6 cm (*N* = 320)
Adm. to 3 cm								
Adm. to 4 cm	2.05 (0.29, 14.50)	1.92 (0.08, 8.33)						
Adm. to 5 cm	3.43 (0.63, 18.55)	4.08 (0.83, 11.75)	1.34 (0.24, 7.51)	1.42 (0.08, 6.42)				
Adm. to 6 cm	4.77 (1.10, 20.63)	5.42 (1.58, 13.33)	2.31 (0.55, 9.66)	2.75 (0.50, 8.08)	0.80 (0.14, 4.72)	0.83 (0.00, 3.58)		
Adm. to 7 cm	5.91 (1.65, 21.17)	6.58 (2.33, 14.75)	2.99 (0.80, 11.18)	3.92 (1.17, 9.67)	1.47 (0.33, 6.54)	1.92 (0.42, 5.58)	0.43 (0.05, 3.50)	0.75 (0.00, 3.50)
Adm. to 8 cm	6.61 (1.97, 22.20)	7.25 (2.83, 15.42)	3.78 (1.19, 11.97)	4.58 (1.58, 10.42)	2.31 (0.61, 8.69)	2.58 (0.75, 6.42)	1.13 (0.22, 5.81)	1.42 (0.25, 4.33)
Adm. to 10 cm	7.55 (2.48, 23.05)	8.25 (3.58, 16.58)	4.63 (1.66, 12.96)	5.58 (2.25, 11.67)	3.43 (1.17, 10.06)	3.58 (1.25, 7.83)	2.19 (0.64, 7.53)	2.42 (0.67, 5.92)
**Parity = 2+**								
Cervical dilatation at:	Adm. at 3 cm *(N* = 231)	Adm. at 3 cm *(N* = 231)	Adm. at 4 cm *(N* = 626)	Adm. at 4 cm *(N* = 626)	Adm. at 5 cm *(N* = 385)	Adm. at 5 cm *(N* = 385)	Adm. at 6 cm *(N* = 414)	Adm. at 6 cm *(N* = 414)
Adm. to 3 cm								
Adm. to 4 cm	2.19 (0.29, 16.32)	2.17 (0.08, 9.75)						
Adm. to 5 cm	3.54 (0.61, 20.75)	4.42 (0.92, 12.92)	1.25 (0.19, 8.14)	1.50 (0.08, 6.50)				
Adm. to 6 cm	4.82 (1.04, 22.38)	5.58 (1.67, 14.25)	2.24 (0.48, 10.48)	2.67 (0.50, 8.00)	0.76 (0.10, 5.80)	0.75 (0.00, 3.33)		
Adm. to 7 cm	5.55 (1.34, 22.96)	6.92 (2.42, 15.75)	3.08 (0.82, 11.52)	3.92 (1.17, 9.75)	1.34 (0.24, 7.46)	1.92 (0.33, 5.50)	0.52 (0.07, 3.65)	0.83 (0.00, 3.58)
Adm. to 8 cm	6.17 (1.63, 23.31)	7.42 (2.92, 16.33)	3.83 (1.18, 12.41)	4.42 (1.50, 10.25)	1.94 (0.40, 9.35)	2.50 (0.67, 6.08)	1.20 (0.26, 5.52)	1.33 (0.25, 4.25)
Adm. to 10 cm	7.24 (2.17, 24.18)	8.25 (3.50, 17.25)	4.71 (1.71, 13.02)	5.33 (2.08, 11.33)	3.07 (0.87, 10.83)	3.33 (1.17, 7.25)	2.39 (0.77, 7.42)	2.25 (0.67, 5.50)

Data presented as median hours (5th, 95th percentiles).

**Abbreviation:** Adm., Admission

**Fig 3:**
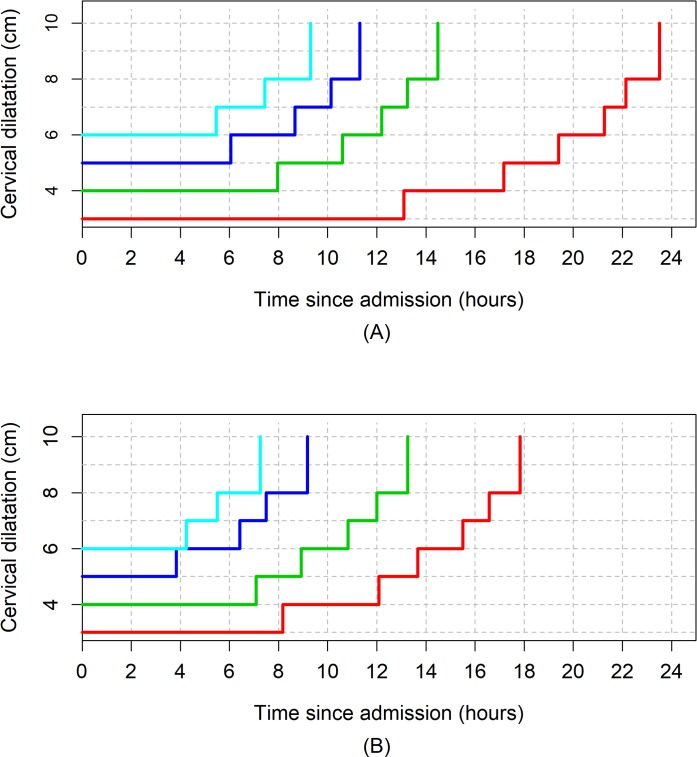
The 95th percentiles of cumulative duration of labour among nulliparous women. (A) Survival analysis. (B) Multistate Markov analysis.

**Fig 4:**
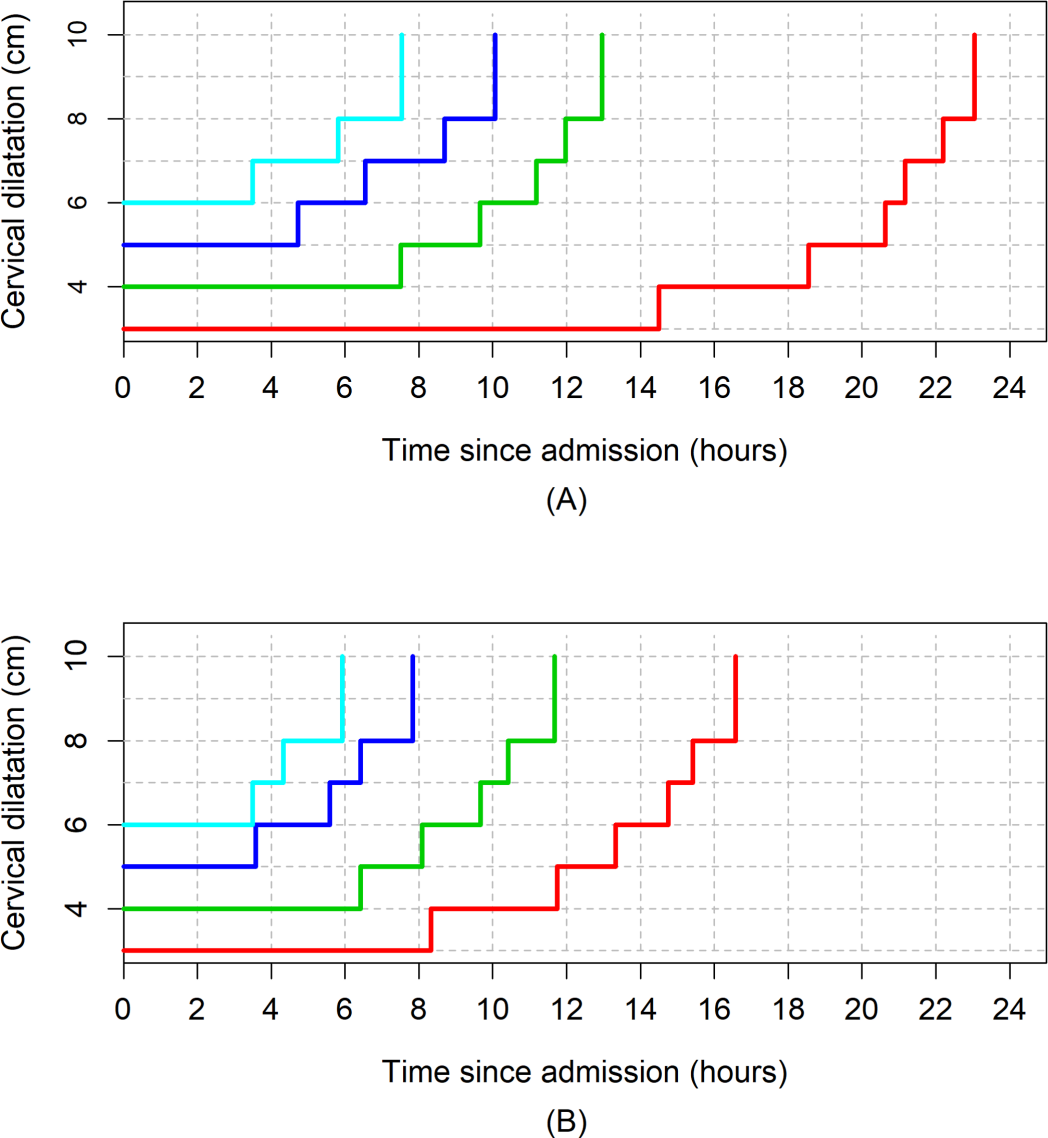
The 95th percentiles of cumulative duration of labour among parity = 1 women. (A) Survival analysis. (B) Multistate Markov analysis.

**Fig 5:**
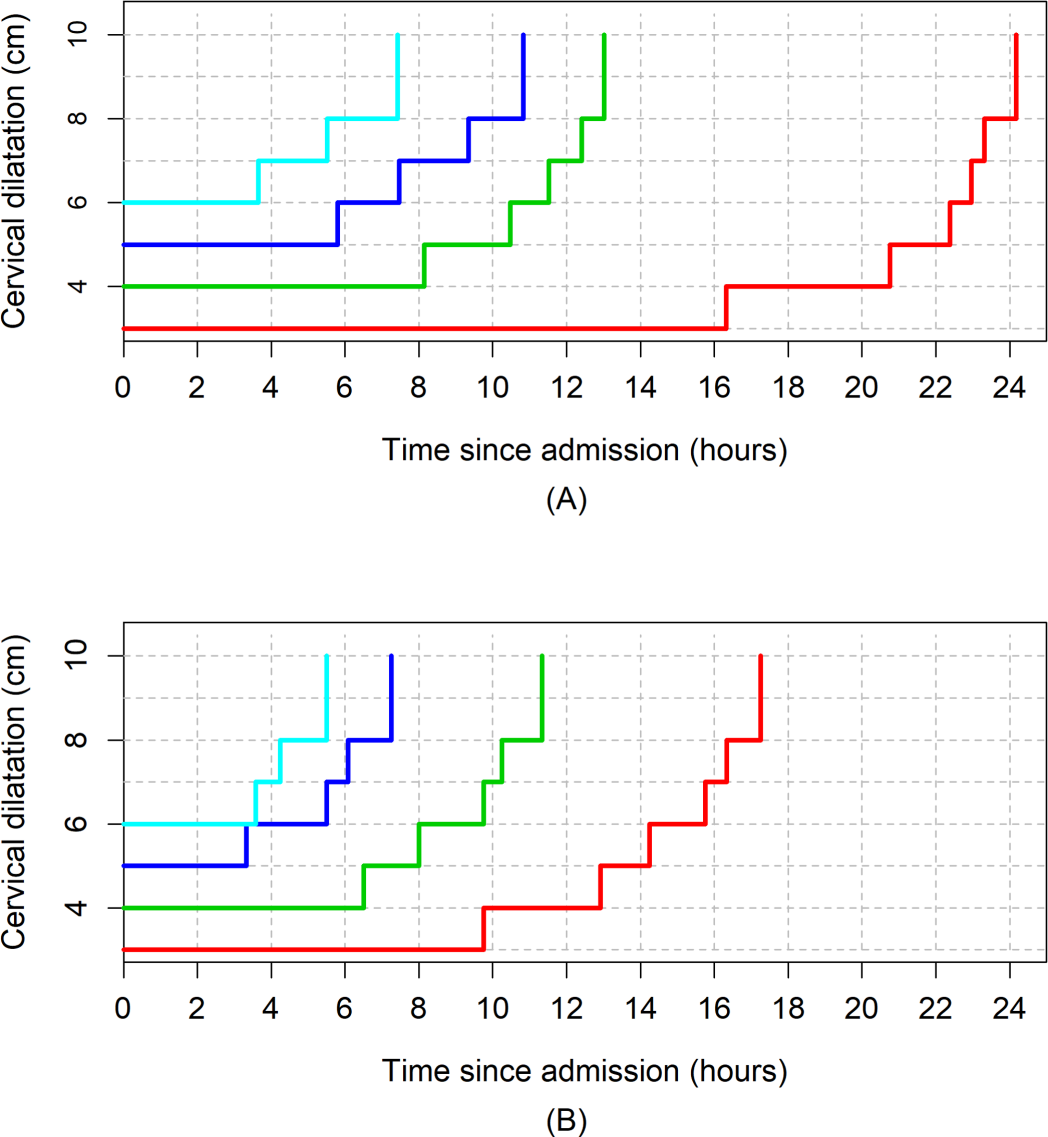
The 95th percentiles of cumulative duration of labour among parity = 2+ women. (A) Survival analysis. (B) Multistate Markov analysis.

Fig 3, Fig 4, and Fig 5 illustrate the 95th percentiles (in Table 3) plotted as connected staircase lines with specified dilatation at admission having its own corresponding line. Based on the dilatation at admission, women falling to the right of these lines (or thresholds) can be regarded as having protracted or unusually slow labour. From the survival analysis data, for example, if a nulliparous woman who was admitted at 4 cm takes longer than 10 hours to reach 6 cm. Likewise, a nulliparous woman admitted at 6 cm can be considered to be experiencing a protracted labour if she takes longer than 7 hours to reach 8 cm or longer than 9 hours to reach 10 cm. The patterns of cumulative labour duration are similar for all parity groups until 6 cm, when the staircase lines become steeper for multiparous compared to nulliparous women.

### Labour progression patterns (excluding women with oxytocin augmentation)

Table 4, Table 5, and Table 6 show the results of the sensitivity analyses of labour progression based on our two analytical approaches. As shown in Table 4, the median, 5th, and 95th percentile times to advance by 1 cm were generally shorter when women who had oxytocin were excluded from the study population. The differences between the median times were generally small, less than half an hour in nearly all cases, and mostly confined to the early part of labour (i.e., between 3 and 5 cm). For nulliparous women, the differences in median times ranged from 5 to 22 minutes, while for parity = 1 and parity = 2+ women, it ranged from 1 to 33 minutes and from less than 1 minute to 27 minutes, respectively. The differences in median times centimetre by centimetre became insignificant as labour advanced.

**Table 4:** Duration of labour from one level of cervical dilatation to the next with and without augmented labours.

**Parity = 0**						
	**All women**	**All women w/o oxytocin**	**Difference in median times**	**All women**	**All women w/o oxytocin**	**Difference in median times**
***N***	**2,100**	**1,300**		**2,166**	**1,300**	
**Cervical dilatation**	**Survival analysis†**	**Survival analysis†**		**Markov model**	**Markov model**	
3–4 cm	2.82 (0.60, 13.33)	2.47 (0.47, 13.14)	0.35	1.83 (0.08, 8.17)	1.75 (0.08, 7.67)	0.08
4–5 cm	1.72 (0.38, 7.83)	1.35 (0.24, 7.57)	0.37	1.58 (0.08, 7.08)	1.67 (0.08, 7.25)	−0.09
5–6 cm	1.19 (0.23, 6.17)	1.01 (0.19, 5.38)	0.18	0.83 (0.00, 3.83)	0.75 (0.00, 3.42)	0.08
6–7 cm	0.66 (0.09, 4.92)	0.46 (0.05, 4.12)	0.20	0.92 (0.00, 4.25)	0.92 (0.00, 4.00)	0.00
7–8 cm	0.25 (0.02, 3.10)	0.16 (0.01, 2.84)	0.09	0.58 (0.00, 2.50)	0.58 (0.00, 2.50)	0.00
8–10 cm	0.87 (0.18, 4.19)	0.76 (0.14; 4.20)	0.11	0.75 (0.00, 3.33)	0.75 (0.00, 3.50)	0.00
**Parity = 1**						
***N***	**1,488**	**1,044**		**1,488**	**1,044**	
**Cervical dilatation**	**Survival analysis†**	**Survival analysis†**		**Markov model**	**Markov model**	
3–4 cm	2.42 (0.41, 14.18)	2.39 (0.50, 11.49)	0.03	1.92 (0.08, 8.33)	1.83 (0.08, 7.92)	0.09
4–5 cm	1.37 (0.25, 7.65)	0.82 (0.11, 6.38)	0.55	1.42 (0.08, 6.42)	1.17 (0.08, 5.33)	0.25
5–6 cm	0.79 (0.13, 4.95)	0.64 (0.10, 4.17)	0.15	0.83 (0.00, 3.58)	0.75 (0.00, 3.17)	0.08
6–7 cm	0.33 (0.03, 3.67)	0.30 (0.03, 3.15)	0.03	0.75 (0.00, 3.50)	0.75 (0.00, 3.42)	0.00
7–8 cm	0.09 (0.00, 2.69)	0.05 (0.00, 2.48)	0.04	0.42 (0.00, 1.83)	0.33 (0.00, 1.75)	0.09
8–10 cm	0.64 (0.11, 3.56)	0.62 (0.12, 3.23)	0.02	0.67 (0.00, 2.92)	0.58 (0.00, 2.58)	0.09
**Parity = 2+**						
***N***	**1,952**	**1,430**		**1,952**	**1,430**	
**Cervical dilatation**	**Survival analysis†**	**Survival analysis†**		**Markov model**	**Markov model**	
3–4 cm	2.35 (0.31, 17.85)	1.90 (0.27, 13.50)	0.45	2.17 (0.08, 9.75)	1.75 (0.08, 7.58)	0.42
4–5 cm	1.18 (0.17, 8.05)	0.83 (0.11, 6.54)	0.35	1.50 (0.08, 6.50)	1.33 (0.08, 5.83)	0.17
5–6 cm	0.79 (0.10, 6.24)	0.61 (0.07, 5.51)	0.18	0.75 (0.00, 3.33)	0.67 (0.00, 2.92)	0.08
6–7 cm	0.31 (0.03, 3.29)	0.25 (0.02, 2.71)	0.06	0.83 (0.00, 3.58)	0.75 (0.00, 3.50)	0.08
7–8 cm	0.17 (0.01, 2.44)	0.16 (0.01, 2.52)	0.01	0.33 (0.00, 1.50)	0.33 (0.00, 1.58)	0.00
8–10 cm	0.68 (0.12, 3.77)	0.69 (0.12, 4.15)	−0.01	0.50 (0.00, 2.50)	0.50 (0.00, 2.42)	0.00

Data presented as median hours (5th, 95th percentiles).

† Survival analysis with complete and interval-censored values

**Abbreviation:** w/o, without.

**Table 5:** Cumulative duration of labour in Para 0, 1, and 2+ based on the cervical dilatation at admission by use of oxytocin augmentation (survival analysis).

**Parity = 0**								
	All women	w/o oxytocin	All women	w/o oxytocin	All women	w/o oxytocin	All women	w/o oxytocin
Cervical dilatation at:	Adm. at 3 cm (*N* = 249)	Adm. at 3 cm (*N* = 158)	Adm. at 4 cm (*N* = 715)	Adm. at 4 cm (*N* = 384)	Adm. at 5 cm (*N* = 316)	Adm. at 5 cm (*N* = 191)	Adm. at 6 cm (*N* = 322)	Adm. at 6 cm (*N* = 201)
Adm. to 3 cm								
Adm. to 4 cm	2.76 (0.58, 13.10)	2.54 (0.48, 13.54)						
Adm. to 5 cm	4.49 (1.17, 17.17)	4.07 (0.90, 18.38)	1.71 (0.37, 7.96)	1.32 (0.25, 7.14)				
Adm. to 6 cm	5.65 (1.65, 19.40)	5.27 (1.36, 20.46)	3.02 (0.86, 10.60)	2.58 (0.66, 10.07)	1.29 (0.28, 6.05)	0.86 (0.15, 4.98)		
Adm. to 7 cm	6.50 (1.99, 21.26)	6.09 (1.67, 22.22)	4.15 (1.41, 12.19)	3.65 (1.11, 11.97)	2.10 (0.51, 8.66)	1.74 (0.40, 7.56)	0.78 (0.11, 5.46)	0.51 (0.06, 4.42)
Adm. to 8 cm	7.19 (2.34, 22.14)	6.81 (2.03, 22.88)	4.97 (1.87, 13.24)	4.51 (1.55, 13.15)	3.06 (0.93, 10.14)	2.69 (0.76, 9.47)	1.76 (0.42, 7.44)	1.46 (0.31, 6.81)
Adm. to 10 cm	8.37 (2.98, 23.51)	7.98 (2.61, 24.42)	5.92 (2.42, 14.48)	5.42 (2.06, 14.25)	4.30 (1.64, 11.30)	4.00 (1.46, 10.97)	2.86 (0.88, 9.30)	2.56 (0.75, 8.75)
**Parity = 1**								
Cervical dilatation at:	Adm. at 3 cm (*N* = 164)	Adm. at 3 cm (*N* = 123)	Adm. at 4 cm (*N* = 491)	Adm. at 4 cm (*N* = 304)	Adm. at 5 cm (*N* = 292)	Adm. at 5 cm (*N* = 211)	Adm. at 6 cm (*N* = 320)	Adm. at 6 cm (*N* = 256)
Adm. to 3 cm								
Adm. to 4 cm	2.05 (0.29, 14.50)	1.98 (0.35, 11.15)						
Adm. to 5 cm	3.43 (0.63, 18.55)	3.24 (0.79 13.34)	1.34 (0.24, 7.51)	0.82 (0.11, 6.25)				
Adm. to 6 cm	4.77 (1.10, 20.63)	4.50 (1.32, 15.35)	2.31 (0.55, 9.66)	1.79 (0.35, 9.19)	0.80 (0.14, 4.72)	0.66 (0.11, 4.03)		
Adm. to 7 cm	5.91 (1.65, 21.17)	5.41 (1.78, 16.46)	2.99 (0.80, 11.18)	2.54 (0.60, 10.84)	1.47 (0.33, 6.54)	1.15 (0.23, 5.73)	0.43 (0.05, 3.50)	0.36 (0.05, 2.68)
Adm. to 8 cm	6.61 (1.97, 22.20)	6.05 (2.08, 17.61)	3.78 (1.19, 11.97)	3.3 (0.95, 11.51)	2.31 (0.61, 8.69)	1.98 (0.49, 8.04)	1.13 (0.22, 5.81)	0.96 (0.19, 4.93)
Adm. to 10 cm	7.55 (2.48, 23.05)	6.92 (2.53, 18.91)	4.63 (1.66, 12.96)	4.08 (1.36, 12.28)	3.43 (1.17, 10.06)	3.17 (1.04, 9.70)	2.19 (0.64, 7.53)	2.03 (0.60, 6.84)
**Parity = 2+**								
Cervical dilatation at:	Adm. at 3 cm	Adm. at 3 cm	Adm. at 4 cm	Adm. at 4 cm	Adm. at 5 cm	Adm. at 5 cm	Adm. at 6 cm	Adm. at 6 cm
	(*N* = 231)	(*N* = 163)	(*N* = 626)	(*N* = 446)	(*N* = 385)	(*N* = 283)	(*N* = 414)	(*N* = 333)
							
Adm. to 3 cm								
Adm. to 4 cm	2.19 (0.29, 16.32)	1.84 (0.27, 12.76)						
Adm. to 5 cm	3.54 (0.61, 20.75)	2.97 (0.52, 16.87)	1.25 (0.19, 8.14)	0.91 (0.12, 6.84)				
Adm. to 6 cm	4.82 (1.04, 22.38)	4.09 (0.89, 18.89)	2.24 (0.48, 10.48)	1.85 (0.37, 9.33)	0.76 (0.10, 5.80)	0.54 (0.06, 5.28)		
Adm. to 7 cm	5.55 (1.34, 22.96)	4.82 (1.18, 19.74)	3.08 (0.82, 11.52)	2.66 (0.66, 10.64)	1.34 (0.24, 7.46)	1.10 (0.17, 7.17)	0.52 (0.07, 3.65)	0.44 (0.06, 3.06)
Adm. to 8 cm	6.17 (1.63, 23.31)	5.46 (1.51, 19.82)	3.83 (1.18, 12.41)	3.46 (1.01, 11.82)	1.94 (0.40, 9.35)	1.67 (0.30, 9.18)	1.20 (0.26, 5.52)	1.06 (0.23, 4.87)
Adm. to 10 cm	7.24 (2.17, 24.18)	6.53 (2.03, 21.01)	4.71 (1.71, 13.02)	4.38 (1.54, 12.41)	3.07 (0.87, 10.83)	2.83 (0.73,10.93)	2.39 (0.77, 7.42)	2.24 (0.71, 7.11)

Data presented as median hours (5th, 95th percentiles).

**Abbreviation:** Adm., Admission

**Table 6:** Cumulative duration of labour in Para 0, 1, and 2+ based on the cervical dilatation at admission by use of oxytocin augmentation (Markov analysis).

**Parity = 0**								
	All women	w/o oxytocin	All women	w/o oxytocin	All women	w/o oxytocin	All women	w/o oxytocin
Cervical dilatation at:	Adm. at 3 cm (*N* = 249)	Adm. at 3 cm (*N* = 158)	Adm. at 4 cm (*N* = 715)	Adm. at 4 cm (*N* = 384)	Adm. at 5 cm (*N* = 316)	Adm. at 5 cm (*N* = 191)	Adm. at 6 cm (*N* = 322)	Adm. at 6 cm (*N* = 201)
Adm. to 3 cm								
Adm. to 4 cm	1.83 (0.08, 8.17)	1.75 (0.08, 7.67)						
Adm. to 5 cm	4.25 (0.83, 12.08)	4.17 (0.83, 11.75)	1.58 (0.08, 7.08)	1.67 (0.08, 7.25)				
Adm. to 6 cm	5.58 (1.67, 13.67)	5.33 (1.58, 13.17)	2.92 (0.58, 8.92)	2.83 (0.58, 8.83)	0.83 (0.00, 3.83)	0.75 (0.00, 3.42)		
Adm. to 7 cm	7.08 (2.50, 15.50)	6.75 (2.42, 15.00)	4.42 (1.33, 10.83)	4.25 (1.25, 10.67)	2.25(0.42, 6.42)	2.08 (0.42, 5.92)	0.92 (0.00, 4.25)	0.92 (0.00, 4.00)
Adm. to 8 cm	7.92 (3.17, 16.58)	7.58 (3.08, 15.83)	5.33 (1.92, 12.00)	5.17 (1.83, 11.67)	3.08 (0.92, 7.50)	2.92 (0.83, 7.08)	1.83 (0.33, 5.5)	1.75 (0.33, 5.25)
Adm. to 10 cm	9.08 (4.00, 17.83)	8.93 (3.92, 17.33)	6.50 (2.67, 13.25)	6.33 (2.58, 13.17)	4.25 (1.58, 9.17)	4.08 (1.50, 8.75)	3.00 (0.92, 7.25)	2.92 (0.83, 7.08)
**Parity = 1**								
Cervical dilatation at:	Adm. at 3 cm (*N* = 164)	Adm. at 3 cm (*N* = 123)	Adm. at 4 cm (*N* = 491)	Adm. at 4 cm (*N* = 304)	Adm. at 5 cm (*N* = 292)	Adm. at 5 cm (*N* = 211)	Adm. at 6 cm (*N* = 320)	Adm. at 6 cm (*N* = 256)
Adm. to 3 cm								
Adm. to 4 cm	1.92 (0.08, 8.33)	1.83 (0.08, 7.92)						
Adm. to 5 cm	4.08 (0.83, 11.75)	3.67 (0.75, 10.58)	1.42 (0.08, 6.42)	1.17 (0.08, 5.33)				
Adm. to 6 cm	5.42 (1.58, 13.33)	4.75 (1.42, 11.92)	2.75 (0.5, 8.08)	2.33 (0.42, 6.92)	0.83 (0.00, 3.58)	0.75 (0.00, 3.17)		
Adm. to 7 cm	6.58 (2.33, 14.75)	6.00 (2.17, 13.33)	3.92 (1.17, 9.67)	3.50 (1.00, 8.50)	1.92 (0.42, 5.58)	1.83 (0.33, 5.33)	0.75 (0.00, 3.50)	0.75 (0.00, 3.42)
Adm. to 8 cm	7.25 (2.83, 15.42)	6.58 (2.58, 14.08)	4.58 (1.58, 10.42)	4.08 (1.42, 9.25)	2.58 (0.75, 6.42)	2.42 (0.67, 6.00)	1.42(0.25, 4.33)	1.42 (0.25, 4.25)
Adm. to 10 cm	8.25 (3.58, 16.58)	7.50 (3.25, 15.17)	5.58 (2.25, 11.67)	5.00 (2.00, 10.33)	3.58 (1.25, 7.83)	3.33 (1.17, 7.33)	2.42 (0.67, 5.92)	2.25 (0.67, 5.67)
**Parity = 2+**								
Cervical dilatation at:	Adm. at 3 cm	Adm. at 3 cm	Adm. at 4 cm	Adm. at 4 cm	Adm. at 5 cm	Adm. at 5 cm	Adm. at 6 cm	Adm. at 6 cm
	(*N* = 231)	(*N* = 163)	(*N* = 626)	(*N* = 446)	(*N* = 385)	(*N* = 283)	(*N* = 414)	(*N* = 333)
							
Adm. to 3 cm								
Adm. to 4 cm	2.17 (0.08, 9.75)	1.75 (0.08, 7.58)						
Adm. to 5 cm	4.42 (0.92, 12.92)	3.75 (0.75, 10.67)	1.50 (0.08, 6.50)	1.33 (0.08, 5.83)				
Adm. to 6 cm	5.58 (1.67, 14.25)	4.75 (1.33, 11.92)	2.67 (0.50, 8.00)	2.33 (0.42, 7.17)	0.75 (0.00, 3.33)	0.67 (0.00, 2.92)		
Adm. to 7 cm	6.92 (2.42, 15.75)	5.92 (2.08, 13.42)	3.92 (1.17, 9.75)	3.58 (1.08, 8.92)	1.92 (0.33, 5.50)	1.75 (0.33, 5.17)	0.83 (0.00, 3.58)	0.75 (0.00, 3.50)
Adm. to 8 cm	7.42 (2.92, 16.33)	6.50 (2.58, 14.00)	4.42 (1.50, 10.25)	4.08 (1.42, 9.50)	2.50 (0.67, 6.08)	2.33 (0.67, 5.83)	1.33 (0.25, 4.25)	1.33 (0.25, 4.25)
Adm. to 10 cm	8.25 (3.50, 17.25)	7.33 (3.17, 14.92)	5.33 (2.08, 11.33)	4.92 (2.00, 10.50)	3.33 (1.17, 7.25)	3.17 (1.17, 6.92)	2.25 (0.67, 5.50)	2.17 (0.67, 5.42)

Data presented as median hours (5th, 95th percentiles).

**Abbreviation:** Adm., Admission

Table 5 and Table 6 show the cumulative duration of labour from the cervical dilatation observed at admission to the next centimetre until 10 cm, excluding women who had oxytocin augmentation. The slightly faster progression of cervical dilatation in the absence of oxytocin augmentation as shown by the sojourn times (in Table 4) is also expressed by the shorter median cumulative duration of labour in all scenarios. For example, considering the cumulative duration of labour for 3 to 10 cm, 4 to 10 cm, 5 to 10 cm, and 6 to 10 cm, the differences in median times were all less than 1 hour regardless of the analysis method used, and the faster progressions were more obvious in women arriving early in labour (i.e., at 3 and 4 cm cervical dilatation).

**Fig 6:**
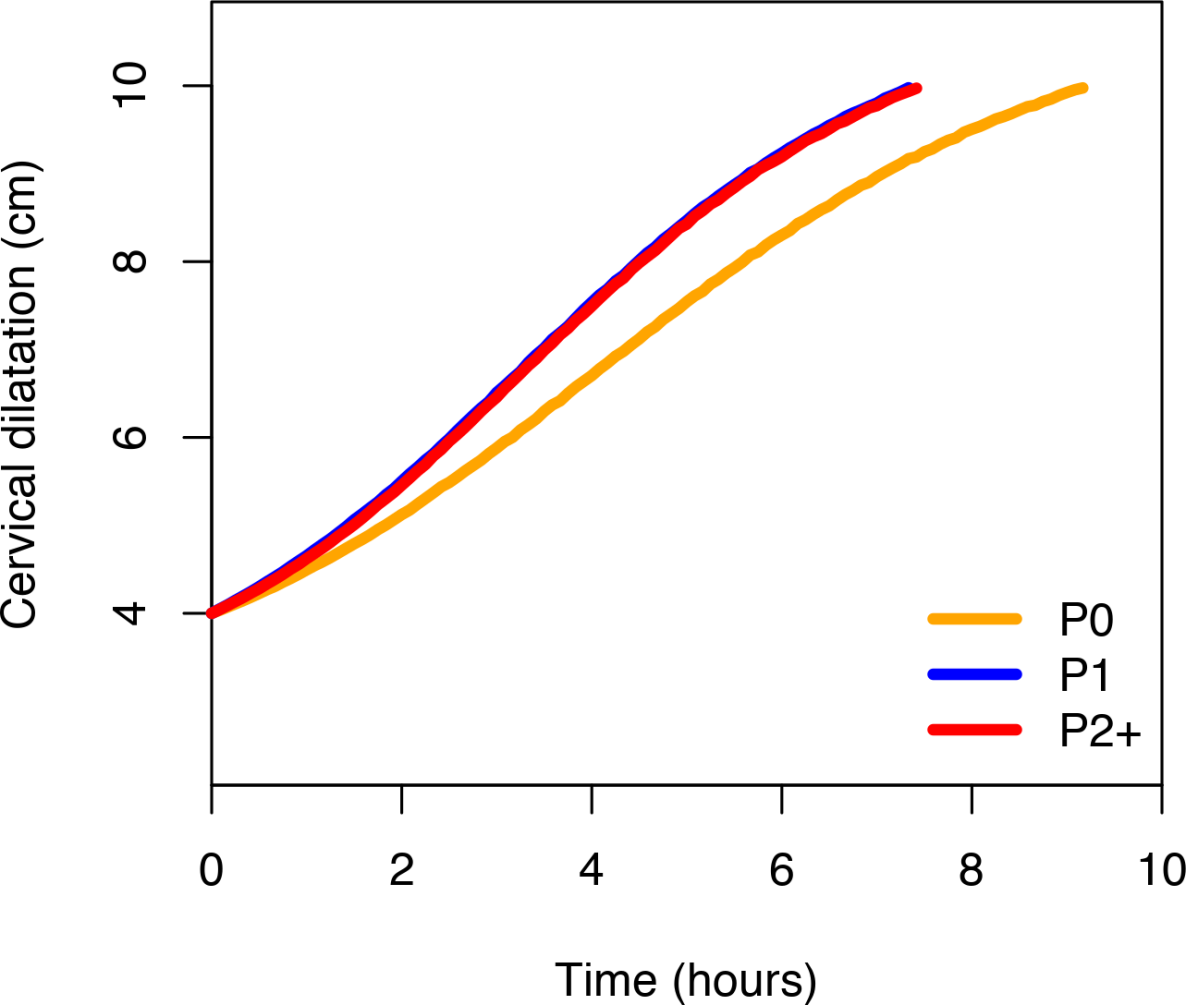
Average labour curves by parity after excluding women with augmented labours. P0, nulliparous women; P1, parity = 1 women; P2+, parity = 2+ women.

**Fig 7:**
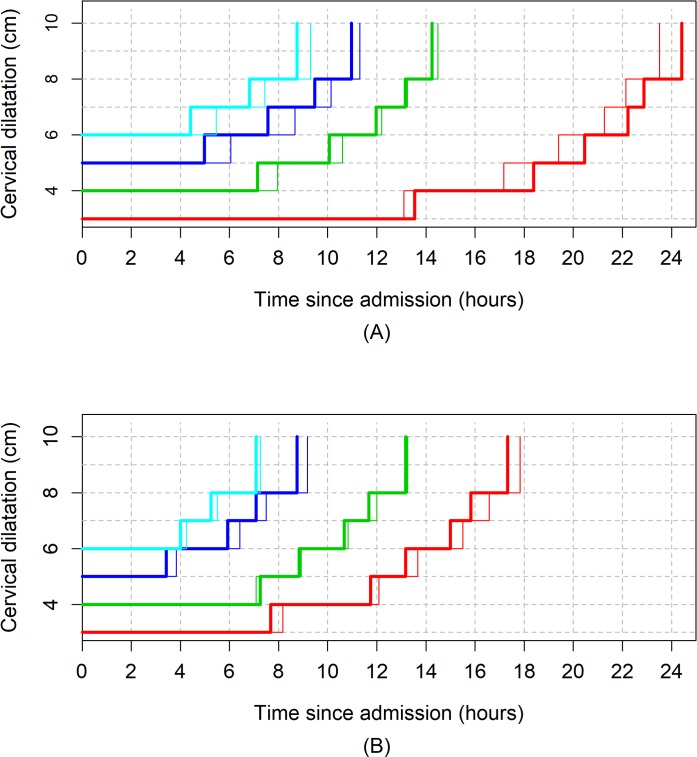
The 95th percentiles of cumulative duration of labour in nulliparous women by augmentation. (A) Survival analysis. (B) Multistate Markov analysis. Thin lines: all women. Thick lines: women with oxytocin augmentation excluded.

**Fig 8:**
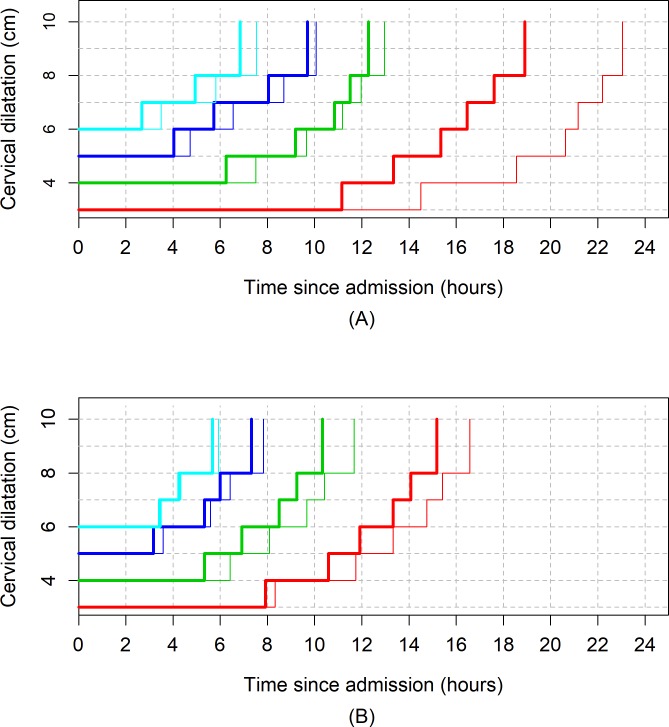
The 95th percentiles of cumulative duration of labour in parity = 1 women by augmentation. (A) Survival analysis. (B) Multistate Markov analysis. Thin lines: all women. Thick lines: women with oxytocin augmentation excluded.

**Fig 9:**
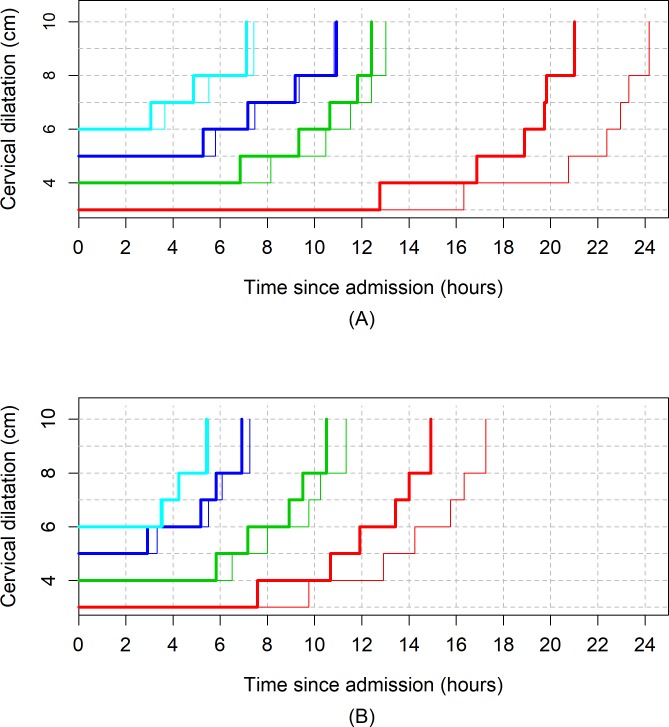
The 95th percentiles of cumulative duration of labour in parity = 2+ women by augmentation. (A) Survival analysis. (B) Multistate Markov analysis. Thin lines: all women. Thick lines: women with oxytocin augmentation excluded.

Fig 6 shows the average labour curves by parity groups after excluding women with oxytocin augmentation. Excluding women who received oxytocin augmentation did not lead to any major change in the pattern or the trajectories of the curves for any parity group. However, the small difference in the labour curves of multiparous groups (as shown in Fig 2) disappeared when women who received oxytocin augmentation were excluded from the analysis. Fig 7, Fig 8, and Fig 9 illustrate the changes in the 95th percentiles (in Table 5 and Table 6) plotted as connected staircase lines for women who received oxytocin augmentation compared to all women. The shorter cumulative labour duration is also reflected in the 95th percentiles for all parity groups regardless of the dilatation at admission, except for nulliparous women admitted at 3 cm, which showed more variability.

## Discussion

### Main findings

Understanding the natural progression of labour presents unique challenges in current obstetric practice. Nevertheless, a gradual shift towards approaches to reduce labour interventions deserves evidence-based information on the upper limits of normal labour to guide practice, especially now that modern analytical methods are available. Contrary to the generally held view, our study shows that in this obstetric population, labour appears to progress more slowly than previously reported [1–3, 27, 28]. The median time needed for the cervix to dilate by 1 cm exceeded 1 hour until dilatation was at least 5 cm in both nulliparous and multiparous women. Labour tended to progress more slowly in the early part of traditional active phase and more rapidly after 6 cm. Considerable variability exists in the distribution of times needed to advance by 1 cm and the duration of labour among women who gave birth vaginally without adverse birth outcomes. For instance, based on 95th percentile thresholds, some nulliparous women took more than 7 hours to advance from 4 to 5 cm, and more than 3 hours to advance from 5 to 6 cm. This pattern of progression was observed irrespective of the analysis method we applied.

While the cumulative duration of labour indicates that a substantial proportion of nulliparous women admitted in labour at 4, 5, and 6 cm achieved full dilatation within an expected time frame if the dilatation rate was ≥ 1 cm/hour, their 95th percentiles show that labour in these women could last up to 14, 11, and 9 hours, respectively, and still lead to a vaginal birth without untoward effects on the mother and baby. Labour could be considerably slow to advance from 3 to 4 cm, and women admitted before 4 cm could have long labours that ultimately end in uncomplicated vaginal birth. Substantial differences exist between actual plots of cervical dilatation over time for individual women and the ‘average labour curves’ derived from our population-level data.

### Strengths and limitations

To our knowledge, this is the first attempt to employ modern statistical and computational mathematical methods to assess the patterns of labour in any African population. We used two analytical approaches to determine labour progression and construct labour curves from the same sample in an attempt to explore whether the resulting patterns are independent of analysis methods. We applied these methods to a relatively large and prospectively collected data set from two sub-Saharan African countries comprising multiethnic groups. However, two main limitations need to be highlighted.

First, our study is prone to selection bias that is inherent in the designs of studies of labour patterns in current obstetric practice [17]. Women excluded from our analysis due to cesarean section during the first or second stage of labour may have a different pattern of labour progression compared with women who had vaginal births. Our perception is that this will not impact our study findings, not only because such women constituted 12% of women in whom vertex delivery was anticipated, but also because the inclusion of women who had cesarean sections as a result of labour dystocia during the first stage or failed operative vaginal birth during the second stage could have biased our results towards even longer labours. Additionally, construction of our labour curves was dependent on using 10 cm as the starting point through a reverse approach, and therefore, it was essential that all women in our study sample reached full dilatation. Nevertheless, the exclusion of women whose labours were induced and those with nonvertex presentation implies that our findings may not be applicable to these women. Our findings also need to be interpreted within the context of non- or low use of epidural anaesthesia and instrumental vaginal birth. As these interventions tend to be associated with slower labours, it is reasonable to assume that their low rates in this population would have biased the current findings towards shorter rather than longer labour duration.

Second is the measurement bias that could have been introduced due to inherent subjectivity in cervical dilatation assessments and a lack of standardization of frequency of pelvic examinations across participating hospitals. Additionally, clinical assessments of cervical dilatation can only be estimates that are rounded up to the nearest centimetre. Given the total number of women analysed for each parity group, any bias from intra- and inter-observer variations is likely to be random with potential impact on the data spread but with minimal effects on the point estimates. However, it is possible that the accuracy of our estimations could have been affected by smaller sample sizes in the subgroups that were used to explore various obstetric characteristics. For example, fewer women in our analysed sample presented to the labour ward at 3 cm or less compared to 4 cm and above in all parity groups. While this reflects the prevailing practices in the study hospitals and most maternity units around the world, it is possible that smaller numbers of women did not permit an equally robust analysis of the passive phase of labour and could have contributed to even wider variability in cervical dilatation profiles during this stage.

### Interpretation

Our findings provide new data from the perspective of a sub-Saharan African population to support the observations reported in similar studies by Zhang [12–14], Suzuki [16], Shi [15], and their colleagues, which suggest that labour progresses more slowly than previously thought. Similar to these studies, our study reveals that the variability of labour progress in a cohort of nulliparous and multiparous women with vaginal birth is greater than generally appreciated. This variability is apparent even in an obstetric population as selected as ours and is independent of our analysis methods, centimetre of cervical dilatation, or cervical dilatation of the woman at admission.

Despite the general similarities in the nulliparous labour progression pattern between our study and those by Zhang [14], Suzuki [16], and Shi [15] et al., there are important differences in the 95th percentiles reported for sojourn times and cumulative durations of labour. Our 95th percentile times indicate that labour can even be slower than what was reported by Zhang [14] and Shi et al. [15], in their American and Chinese populations, respectively, but not as long as Suzuki et al. [16] reported for Japanese women. While this may be due to the differences in the methods for analysing labour progression, a more logical explanation is the heterogeneity in these study populations in terms of labour interventions and demographic characteristics. For instance, oxytocin augmentation among nulliparous women was more common in the US population (47%) studied by Zhang et al. [14] and our study population (40%), but infrequent (6.5%) in the Japanese population studied by Suzuki et al. [16].

The described patterns of labour progress from our study deviate substantially from what Friedman’s curve indicates [1–3]. The classic sigmoidal pattern was not observed in our average labour curves. This may be due to the fact that the majority of the women in our study were not admitted early enough in labour to substantially reflect the pattern of the passive phase of labour and because of the lack of documented assessment of 9-cm dilatation in our cohort, which precluded exploration of any deceleration between 9 and 10 cm. In his series of 500 nulliparous women [2], Friedman used the mean values of the four separate phases of individually plotted sigmoid curves to derive the mean labour curve and reported 1.2 cm/hour as the minimum value of ‘phase of maximum slope’ based on the 95th percentile point on the distribution curve. The nulliparous average curves from our cohort are less steep, and the 95th percentile values from one level of dilatation to the next during the traditional active phase yielded median rates between 0.1 and 0.5 cm/hour between 4 and 10 cm. It remains unclear to what extent an average labour curve depicts the variability associated with individual women’s labour progress, and its value in clinical practice is becoming increasingly questioned. The differences illustrated by the video displays of individual labour profiles, compared to the average labour curves for this cohort, indicate how unreliable a population average curve is in representing an individual woman’s labour progression profile.

In an attempt to overcome the shortcomings of Friedman’s labour curves, Zhang et al. [12] proposed the use of repeated measures analysis with polynomial modelling as a superior method for constructing labour curves, given its flexibility to fit labour data. Other investigators using the same statistical method have confirmed a similar pattern of labour curves published by Zhang et al [12–14]. However, we found that the polynomial model was not appropriate for our data, as it presents a behaviour that is incompatible with labour curve modelling. Rather, we applied multistate Markov modelling to overcome the unpredictable nature of cervical dilatation [29], since its models can accommodate the inherent randomness in cervical dilatation over time [30] and it has the advantage of providing a better representation of real life scenarios from more angles by including empirical observations. We also applied a nonlinear mixed model because of its advantages in terms of interpretability, parsimony, and validity [31]. Although the curves obtained from our nonlinear mixed models are similar to those constructed through polynomial models by previous authors [12, 14, 15], they should be interpreted with caution, as the model appears dependent on extrapolation beyond the normal range of observations for women in the sample.

An interesting finding in our study is the median cumulative duration of labour (e.g., from 4 to 10, 5 to 10, and 6 to 10 cm), which, when considered linearly, suggests that the cervix was dilating at ≥ 1 cm/hour. However, such interpretation hides the nonlinearity of labour progression patterns for most women and does not account for slower progress at the beginning of the traditional active phase and faster progress when active phase is advanced. This implies that some women within the 95th percentile boundary as shown in our study will be categorised as having protracted labour if current labour standards were applied. For instance, a woman with reassuring maternal and fetal conditions who remains at 4 cm for 4 hours may be subjected to oxytocin augmentation when she could still be within her normal limits before advancing to 5 cm. Application of interventions too soon when a woman is still within the boundaries of her normality probably accounts for escalating rates of interventions to expedite labour globally.

One subject of debate in the analysis of labour progression patterns in contemporary practice is the potential impact of oxytocin augmentation on observed labour patterns. A widely held view is that the inclusion of women with augmented labour is likely to produce faster labour progression profiles, and the restriction of analysis to women without labour augmentation will generate labour profiles that reflect natural labour progression. However, we found the contrary, as the exclusion of women with augmented labours from our study population resulted in generally faster labour progression patterns. Although unexpected, this finding was not surprising, as it reflects the impact of Friedman’s original curves and their derivative tools on labour management even today. Women with augmented labours were those assessed by labour attendants as having slower than normal progression based on a preconceived expectation of 1 cm/hour cervical dilatation. Therefore, their exclusion from the analysed study population leaves a highly selected population of women whose labour progression, by the assessment of the labour attendants, conformed to this preconceived expectation and did not require labour augmentation. While the overall clinical implications of the altered progression in terms of labour duration are minimal, our findings support the inclusion of women with augmented labours in the analysis of labour progression in the context where use of oxytocin is the norm so as to facilitate applicability of their findings.

### Conclusions and recommendations

We acknowledge that the described labour patterns from this cohort may be related to the demographic characteristics and prevailing clinical practices in our study settings. Nevertheless, a number of clear messages emerged from our study. First, population average labour curves are at best estimates that may not truly reflect the variability associated with labour progress and could potentially misclassify individual women. It appears that average labour curves are dependent on the underlying assumptions and principles governing the statistical methods from which they are derived. We conclude that population average labour curves are merely useful for illustrative purposes.

Secondly, our labour progression data clearly demonstrate that a minimum cervical dilatation rate of 1 cm/hour throughout the period traditionally described as active phase may be unrealistically fast for some women and should therefore not be universally applied as a threshold for identifying abnormally progressing labour. Likewise, for most nulliparous and multiparous women, labour may not accelerate until a threshold of at least 5 cm is reached. The implication is that a cervical dilatation rate slower than 1 cm/hour throughout the first stage of labour, especially before 5 cm, should not be an indication for interventions to expedite labour provided maternal and fetal vital signs and other observations are normal. It would be useful for labour care providers to consider the upper boundaries reported in this cohort when reviewing whether an intervention is justified. It is important to note, however, that the presented percentile values are insufficient to define abnormal labour that requires interventions to avert adverse outcomes. As this is a selected sample of women without adverse birth outcomes, we cannot conclude from the current analysis whether women with cervical dilatation progressing beyond our percentile values (or other specific boundaries) have comparatively higher risk of adverse birth outcomes. As cervical dilatation is a reflection of a complex interaction of biological, physical, and psychological factors during the course of labour, it is imperative that women with a suspicion of protracted labour be carefully evaluated to exclude developing complications (e.g., cephalopelvic disproportion) and to ensure that the woman’s physical and emotional needs are being met. In the absence of any problems other than a slower than expected cervical dilatation (i.e., 1 cm/hour), it is in the interest of the woman that expectant, supportive, and woman-centred labour care is continued.

We propose that averaged lines or curves are not used for decision-making in the management of labour for individual women. Efforts should focus on developing individualised (or personalised) labour management algorithms that optimize woman-centred health outcomes. Decision-analysis models and machine learning technologies that are available today can assist in achieving this objective.

## Supporting information

S1 STROBE Checklist (DOC)

S1 Fig States and matrix of possible transitions of cervical dilatation. (a) Schematic representation of possible states from 2 cm to 10 cm of cervical dilatation until birth (absorbing state). (b) Matrix representation of all possible transitions between states of cervical dilatation.
(TIF)

S2 Fig 3D graphical illustration of transition (matrix) model. The temporal evolution of the distribution representing the theoretical cohort entering labour at 2 cm of cervical dilation. Example of graphical representation of the transition (matrix) model for a simple case study where each state (2, 3, 4, 5, 6, 7, 8, 10) is modelled as the possible next cervical dilatation until the delivery state (D). Simulation was for a period cycle of 1 hour between transitions for the sake of simplicity.
(TIF)

S3 Fig Average labour curves by parity based on nonlinear mixed models. P0, nulliparous women; P1, parity = 1 women; P2+, parity = 2+ women.
(TIFF)

S1 Video Individual plots of cervical dilatation, average labour curve (from Markov models), and alert line for nulliparous women. (MP4)

S2 Video Individual plots of cervical dilatation, average labour curves (from Markov models), and alert line for multiparous women. (MP4)

S3 Video Individual plots of cervical dilatation, average labour curve (from nonlinear mixed models), and alert line for nulliparous women. (MP4)

S4 Video Individual plots of cervical dilatation, average labour curves (from nonlinear mixed models), and alert line for multiparous women. (MP4)

S1 Data Data set. (CSV)

S2 Data Data dictionary. (XLSX)

## Abbreviations

BOLD: Better Outcomes in Labour Difficulty
RP2: Review Panel on Research Projects
