# Supplementary material for: Progression of the first stage of spontaneous labour: A prospective cohort study in two sub-Saharan African countries
Source: PLoS Med. 2018 Jan 16;15(1):e1002492. doi: 10.1371/journal.pmed.1002492 (PMC5770022; doi:10.1371/journal.pmed.1002492)
Supplement: S1 STROBE Checklist — (DOC) [file pmed.1002492.s001.doc]

STROBE Statement—Checklist of items that should be included in reports of ***cohort studies***

|  | Item No | Recommendation |
| --- | --- | --- |
| **Title and abstract** | 1 | (*a*) Indicate the study’s design with a commonly used term in the title or the abstract [**See the title of the manuscript where “a prospective cohort study …” is stated, and in Methods, paragraph 1, of the Abstract**] |
|  |  | (*b*) Provide in the abstract an informative and balanced summary of what was done and what was found  **[See Abstract, Methods and Findings]** |
| Introduction | | |
| Background/rationale | 2 | Explain the scientific background and rationale for the investigation being reported **[See Introduction, paragraphs 2 and 3, and the excerpts below]:**  Since the late 1990s and early 2000s, there is increasing evidence to suggest that the descriptions of the relationship between the duration of first stage of labour and cervical dilatation patterns [*as published by Friedman in the 1950s and 1960s*]; and the definitions of labour dystocia as earlier described may not be appropriate [references 12-16].  In addition, newer research has taken advantage of methodological advancements in computational techniques to address the limitations of studying labour progression and constructing labour curves in the 1950s and 1960s [reference 17]. While these advancements have led to novel findings and new guidance on labour care [reference 18], they are also a subject of intense debate [references 19-21].  While contemporary labour curves have been published for White, Hispanic, and Asian obstetric populations [references 14-16], no modern labour curves exists for sub-Saharan African women. |
| Objectives | 3 | State specific objectives, including any prespecified hypotheses.  **[See Introduction, paragraph 4, and the excerpt below]**:  As part of the World Health Organization’s BOLD (“Better Outcomes in Labour Difficulty”) project which aimed to develop an innovative and effective labour monitoring-to-action tool [reference 23], we examined the patterns of labour progression in a prospective cohort of women in Nigeria and Uganda who gave birth vaginally without adverse birth outcomes following a spontaneous labour onset. |
| Methods | | |
| Study design | 4 | Present key elements of study design early in the paper.  **[See Methods, paragraph 2, and the excerpt below]**:  In brief, this was a prospective, multicentre, cohort study of women admitted for vaginal birth with single live fetuses during early first stage of labour across thirteen hospitals in Nigeria and Uganda. |
| Setting | 5 | Describe the setting, locations, and relevant dates, including periods of recruitment, exposure, follow-up, and data collection  **[See Methods, paragraphs 2 and 3** (*Design, Setting and Population sub-sections*)**]** |
| Participants | 6 | (*a*) Give the eligibility criteria, and the sources and methods of selection of participants. Describe methods of follow-up  **[See Methods, paragraph 5** (*Study Procedures)*, **Fig 1** (*sample selection flow chart*) **and the excerpt below]**:  The current study used information on maternal baseline and admission characteristics, repeated assessments of cervical dilatation over time, maternal and neonatal characteristics throughout labour and perinatal outcome data. This analysis was focused on describing the labour patterns of women without adverse birth outcomes and not on determining correlation to clinical outcomes. From a total of 8957 singleton births with consistent time records in the database, we restricted our analysis to examine labour progression to 5606 women on the basis of the following inclusion criteria (Fig 1): |
|  |  | (*b*) For matched studies, give matching criteria and number of exposed and unexposed **[N/A]** |
| Variables | 7 | Clearly define all outcomes, exposures, predictors, potential confounders, and effect modifiers. Give diagnostic criteria, if applicable  **[See Methods, paragraph 6** *(Data analysis)***,** **and the excerpt below]:**  In the first approach, we performed survival analyses to estimate the time it took to progress from one level of cervical dilatation to the next (called “sojourn time”) (i.e. from 3 to 4 cm, 4 to 5 cm, 5 to 6 cm until full dilatation (10 cm) […]We used the same approach to derive the cumulative duration of labour for women presenting at different cervical dilatations (3 cm, 4 cm, 5 cm, and 6 cm) to evaluate any potential differences in the patterns of labour progression. |
| Data sources/ measurement | 8* | For each variable of interest, give sources of data and details of methods of assessment (measurement). Describe comparability of assessment methods if there is more than one group  **[See Methods, paragraph 5, and the excerpt below]:**  From a total of 8957 singleton births with consistent time records in the database, we restricted our analysis to examine labour progression to 5606 women on the basis of the following inclusion criteria (Fig. 1: sample selection process) |
| Bias | 9 | Describe any efforts to address potential sources of bias  **[See Methods, paragraphs 7 and 10, and the excerpts below]:**  …We [independently] applied a multistate Markov modelling technique to examine the labour progression patterns in the same sample.  In order to assess the influence of oxytocin augmentation on the described labour patterns, we applied the two approaches to perform sensitivity analyses comparing labour progression patterns of all women with that of a population excluding women with oxytocin augmentation (i.e. our entire study population versus study population excluding women with augmented labours). |
| Study size | 10 | Explain how the study size was arrived at  **[See Methods, paragraph 5, and the excerpt below]:**  From a total of 8957 singleton births with consistent time records in the database, we restricted our analysis to examine labour progression to 5606 women on the basis of the following inclusion criteria (Fig 1): term births (between 37 weeks and 0 days and 41 weeks and 6 days) with vertex presentation and spontaneous labour onset. We excluded women who had labour induction, previous uterine scar, or intrapartum caesarean section. To examine the labour patterns in women with normal perinatal outcomes, we excluded women whose labour resulted in severe adverse outcomes which was defined as occurrence of any of the following: stillbirth, early neonatal death, neonatal use of anticonvulsant, neonatal cardio-pulmonary resuscitation, 5-minute Apgar score < 6, maternal death or organ dysfunction associated labour dystocia and uterine rupture. Furthermore, we excluded women who gave birth to neonates with severe congenital malformation and those with fewer than two cervical dilatation assessments during the first stage of labour (since a single data point cannot be used to generate labour pattern for the individual woman). |
| Quantitative variables | 11 | Explain how quantitative variables were handled in the analyses.  **[See Methods, paragraphs 6-9** *(Data analysis)***]**  If applicable, describe which groupings were chosen and why  **[See Methods, paragraphs 6** *(Data analysis )****,*****and excerpt below]**:  We grouped women in the selected sample into three parity groups (0, 1, and 2+) to illustrate any differences in labour patterns according to parity. |
| Statistical methods | 12 | (*a*) Describe all statistical methods, including those used to control for confounding **[See Methods, paragraphs 6-9** *(Data analysis)***]** |
|  |  | (*b*) Describe any methods used to examine subgroups and interactions **[See Methods, paragraphs 6-9** *(Data analysis)***]** |
|  |  | (*c*) Explain how missing data were addressed **[See data presented as “Unknown” in Table 1]** |
|  |  | (*d*) If applicable, explain how loss to follow-up was addressed **[N/A]** |
|  |  | (*e*) Describe any sensitivity analyses  **[See Methods, paragraph 9, and the excerpt below]**:  In order to assess the influence of oxytocin augmentation on the described labour patterns, we applied the two approaches to perform sensitivity analyses comparing labour progression patterns of all women with that of a population excluding women with oxytocin augmentation (i.e. our entire study population versus study population excluding women with augmented labours). |
| Results | | |
| Participants | 13* | (a) Report numbers of individuals at each stage of study—eg numbers potentially eligible, examined for eligibility, confirmed eligible, included in the study, completing follow-up, and analysed  **[See Fig 1 for Sample selection flow chart]** |
|  |  | (b) Give reasons for non-participation at each stage  **[See Fig 1 for Sample selection flow chart]** |
|  |  | (c) Consider use of a flow diagram  **[See Fig 1 for Sample selection flow chart]** |
| Descriptive data | 14* | (a) Give characteristics of study participants (eg demographic, clinical, social) and information on exposures and potential confounders  **[See Table 1]** |
|  |  | (b) Indicate number of participants with missing data for each variable of interest  **[See Table 1]** |
|  |  | (c) Summarise follow-up time (eg, average and total amount)  **[See Methods, paragraph 4, and the excerpt below]**:  …trained research nurses prospectively extracted detailed information on socio-demographic, anthropometric, obstetric and medical characteristics of study participants at hospital admission, multiple assessments for labour monitoring and interventions performed throughout the first and second stages of labour, and maternal and neonatal labour outcomes. […] Data collection was limited to hospital stay of the mother and baby and there was no follow-up after hospital discharge. |
| Outcome data | 15* | Report numbers of outcome events or summary measures over time  **[N/A]** |
| Main results | 16 | (*a*) Give unadjusted estimates and, if applicable, confounder-adjusted estimates and their precision (eg, 95% confidence interval). Make clear which confounders were adjusted for and why they were included  **[N/A]** |
|  |  | (*b*) Report category boundaries when continuous variables were categorized |
|  |  | (*c*) If relevant, consider translating estimates of relative risk into absolute risk for a meaningful time period  **[N/A]** |
| Other analyses | 17 | Report other analyses done—eg analyses of subgroups and interactions, and sensitivity analyses  **[See Tables 4, 5 and 6, and the excerpts below]**  As shown in Table 4, the median, 5th and 95th percentile times to advance by 1 cm were generally shorter when women who had oxytocin were excluded from the study population. The differences between the median times were generally small, less than half an hour in nearly all cases, and mostly confined to the early part of labour (between 3 and 5 cm). For nulliparous women, the difference in median times ranged from 5 to 22 minutes, while for parity=1 and parity=2+, it ranged from 1 to 33 minutes and from less than 1 to 27 minutes, respectively. The difference becomes insignificant as labour advanced.  Table 5 and Table 6 show the cumulative duration of labour from the cervical dilatation observed at admission to the next until 10 cm excluding women who had oxytocin augmentation. The slightly faster progression of cervical dilatation in the absence of oxytocin augmentation as shown by the sojourn times (in Table 4) is also expressed by the shorter median cumulative duration of labour in all scenarios. For example, considering the cumulative duration of labour for 3 to 10 cm, 4 to 10 cm, 5 to 10 cm and 6 to 10 cm, the differences in median times were all less than 1 hour regardless of the analysis method used, and the faster progressions were more obvious in women arriving early in labour (i.e. at 3 and 4 cm cervical dilatation). |
| Discussion | | |
| Key results | 18 | Summarise key results with reference to study objectives  **[See Discussion, paragraph 1** (*Main Findings*)**, and the excerpts below]:**  Contrary to the generally held view, our study shows that in this obstetric population, labour appears to progress more slowly than previously reported.[…] The median time needed for the cervix to dilate by 1 cm exceeded 1 hour until dilatation was at least 5 cm in both nulliparous and multiparous women. Labour tended to progress more slowly in the early part of traditional active phase and more rapidly after 6 cm. Considerable variability exists in the distribution of times needed to advance by 1 cm and the duration of labour among women who gave birth vaginally without adverse perinatal outcomes. |
| Limitations | 19 | Discuss limitations of the study, taking into account sources of potential bias or imprecision. Discuss both direction and magnitude of any potential bias  **[See Discussion. Paragraph 3** (*Strengths and Limitations*)**, and the excerpts below]**:  First, our study is prone to selection bias that is inherent in the designs of studies of labour patterns in current obstetric practice [reference 17]. Women excluded from our analysis due to caesarean section during the first or second stage of labour may have a different pattern of labour progression compared with women who had vaginal births. Our perception is that this will not impact on our study findings, not only because such women constituted 12% of women in whom vertex delivery was anticipated, but also because the inclusion of women who had caesarean section as a result of labour dystocia during the first stage or failed operative vaginal birth during second stage could have biased our results towards even longer labours. |
| Interpretation | 20 | Give a cautious overall interpretation of results considering objectives, limitations, multiplicity of analyses, results from similar studies, and other relevant evidence  **[See Discussion, paragraphs 6-10]** |
| Generalisability | 21 | Discuss the generalisability (external validity) of the study results  **[See Discussion, paragraph 7, and the excerpts below]:**  Despite the general similarities in the nulliparous labour progression pattern between our study and Zhang [reference 14], Suzuki [reference 16], and Shi [reference 15] et al., there are important differences in the 95th percentiles reported for sojourn times and cumulative duration of labour. Our 95th percentile times indicate that labour can even be slower than what was reported by Zhang [reference 14] and Shi et al. [reference 15], in their American and Chinese populations, respectively, but not as long as Suzuki et al. [16] reported for Japanese women. While this may be due to the differences in the methods for analysing labour progression, a more logical explanation is the heterogeneity in these study populations in terms of labour interventions and demographic characteristics. |
| Other information | | |
| Funding | 22 | Give the source of funding and the role of the funders for the present study and, if applicable, for the original study on which the present article is based  **[See Funding section]** |

*Give information separately for exposed and unexposed groups.

**Note:** An Explanation and Elaboration article discusses each checklist item and gives methodological background and published examples of transparent reporting. The STROBE checklist is best used in conjunction with this article (freely available on the Web sites of PLoS Medicine at http://www.plosmedicine.org/, Annals of Internal Medicine at http://www.annals.org/, and Epidemiology at http://www.epidem.com/). Information on the STROBE Initiative is available at http://www.strobe-statement.org.
