# Supplementary figures and images for: Progression of the first stage of spontaneous labour: A prospective cohort study in two sub-Saharan African countries
Source: PLoS Med. 2018 Jan 16;15(1):e1002492. doi: 10.1371/journal.pmed.1002492 (PMC5770022; doi:10.1371/journal.pmed.1002492)

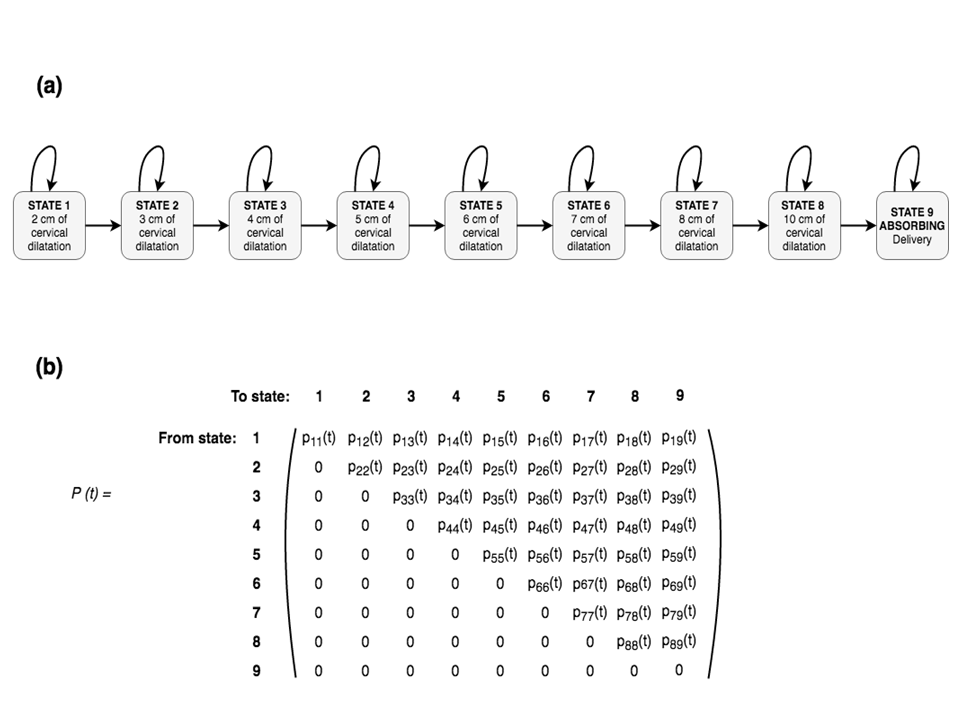

Supplement: S1 Fig — (a) Schematic representation of possible states from 2 cm to 10 cm of cervical dilatation until birth (absorbing state). (b) Matrix representation of all possible transitions between states of cervical dilatation. (TIF) [file pmed.1002492.s002.tif]

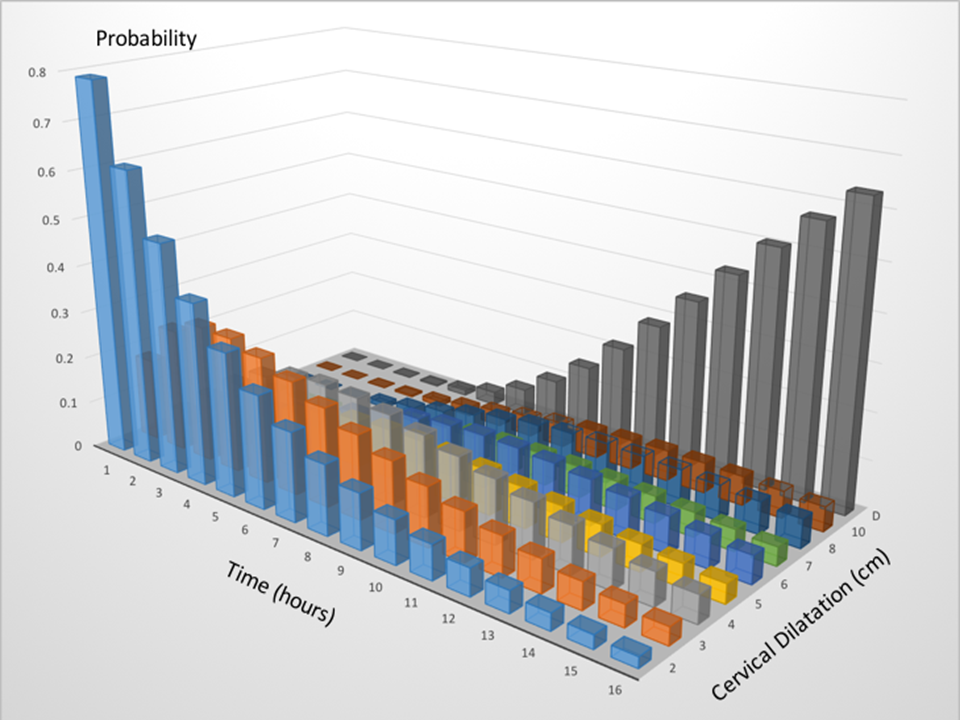

Supplement: S2 Fig — The temporal evolution of the distribution representing the theoretical cohort entering labour at 2 cm of cervical dilation. Example of graphical representation of the transition (matrix) model for a simple case study where each state (2, 3, 4, 5, 6, 7, 8, 10) is modelled as the possible next cervical dilatation until the delivery state (D). Simulation was for a period cycle of 1 hour between transitions for the sake of simplicity. (TIF) [file pmed.1002492.s003.tif]

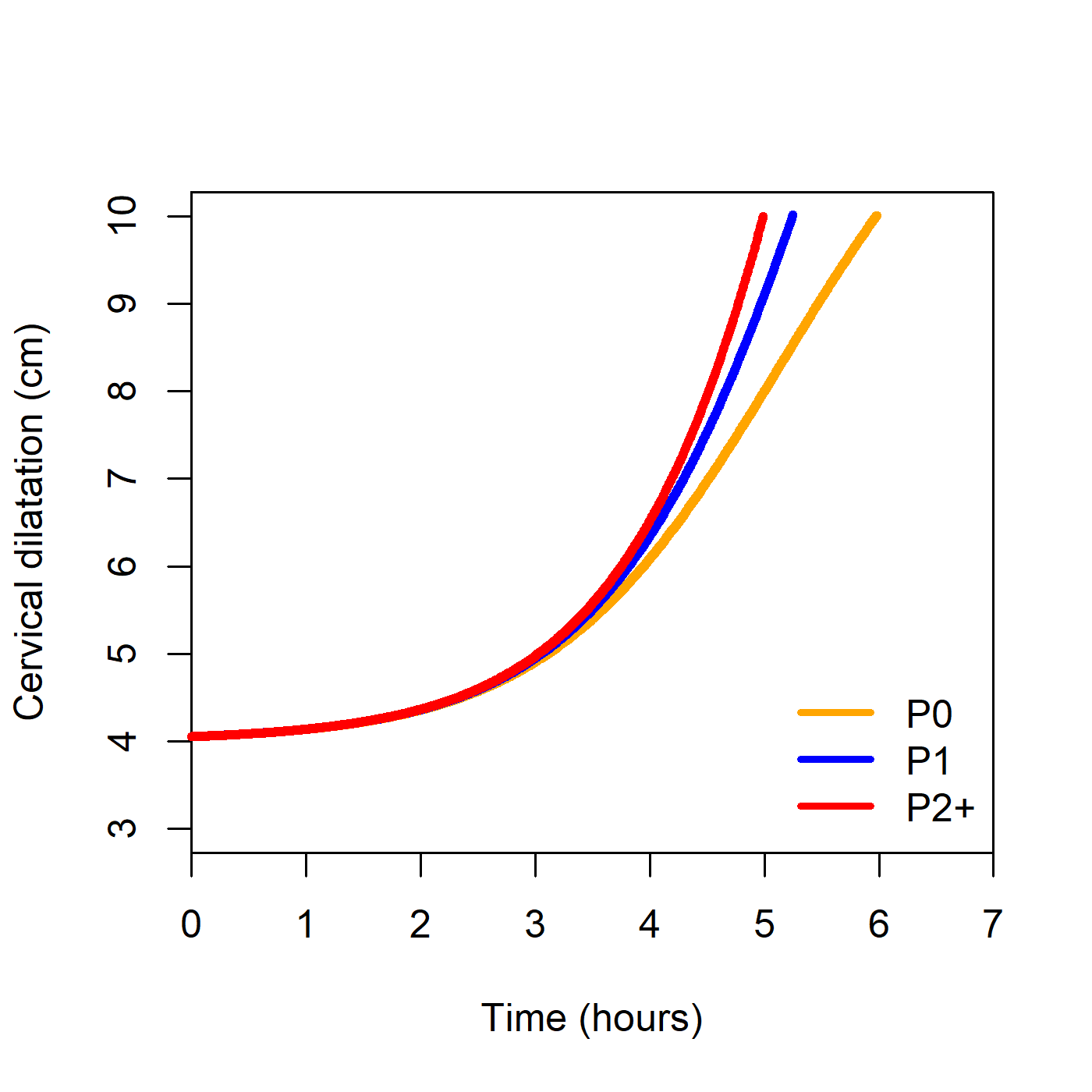

Supplement: S3 Fig — P0, nulliparous women; P1, parity = 1 women; P2+, parity = 2+ women. (TIFF) [file pmed.1002492.s004.tiff]
